# Highly active nanostructured CoS_2_/CoS heterojunction electrocatalysts for aqueous polysulfide/iodide redox flow batteries

**DOI:** 10.1038/s41467-019-11176-y

**Published:** 2019-07-29

**Authors:** Dui Ma, Bo Hu, Wenda Wu, Xi Liu, Jiantao Zai, Chen Shu, Tsegaye Tadesse Tsega, Liwei Chen, Xuefeng Qian, T. Leo Liu

**Affiliations:** 1 Shanghai Electrochemical Energy Devices Research Center, School of Chemistry and Chemical Engineering and State Key Laboratory of Metal Matrix Composites, Shanghai Jiao Tong University, 200240 Shanghai, P. R. China; 2 The Department of Chemistry and Biochemistry, Utah State University, Logan, UT 84322 USA; 3 Suzhou Institute of Nano-Tech and Nano-Bionics (SINANO), Chinese Academy of Sciences, 215123 Suzhou, P. R. China

**Keywords:** Nanoparticles, Batteries

## Abstract

Aqueous polysulfide/iodide redox flow batteries are attractive for scalable energy storage due to their high energy density and low cost. However, their energy efficiency and power density are usually limited by poor electrochemical kinetics of the redox reactions of polysulfide/iodide ions on graphite electrodes, which has become the main obstacle for their practical applications. Here, CoS_2_/CoS heterojunction nanoparticles with uneven charge distribution, which are synthesized in situ on graphite felt by a one-step solvothermal process, can significantly boost electrocatalytic activities of I^−^/I_3_^−^ and S^2−^/S_x_^2−^ redox reactions by improving absorptivity of charged ions and promoting charge transfer. The polysulfide/iodide flow battery with the graphene felt-CoS_2_/CoS heterojunction can deliver a high energy efficiency of 84.5% at a current density of 10 mA cm^−2^, a power density of 86.2 mW cm^−2^ and a stable energy efficiency retention of 96% after approximately 1000 h of continuous operation.

## Abstract (web summary)

Polysulfide/iodide redox flow batteries are promising due to low cost and high-solubility components, but are limited by energy efficiency and power density. Here the authors fabricate heterojunction electrocatalysts to achieve improved performance in a polysulfide/iodide redox flow battery.

## Introduction

Energy storage technologies are crucial for effectively utilizing intermittent renewable resources like solar and wind^1–3^. Inorganic or organic redox flow batteries (RFBs) have been explored and advocated as a prospective technology for grid-scale energy storage in virtue of their design flexibility in decoupling power and energy, high power performance, and ease of scale-up^4–23^. Nowadays, all-vanadium RFBs represent the current state-of-the-art, but their system price is near 4-fold higher than the price targets outlined by the Department of Energy of U.S., $100^24^. Besides developing abundant redox organic charge storage materials^3^, it remains highly technologically and economically attractive to advance the flow battery chemistries of abundant and low cost redox active inorganic compounds such as iron salts, halides, and sulfides ^2^.

1 $${\mathrm{Cathode}}:3{\mathrm{I}}^ - - 2e^ - \leftrightarrow {\mathrm{I}}_3^ - E^\theta = 0.54\;{\mathrm{V}}\;{\mathrm{vs}}\;{\mathrm{SHE}}$$

2 $${\mathrm{Anode}}:{\mathrm{S}}_4^{2 - } + 2e^ - \leftrightarrow 2{\mathrm{S}}_2^{2 - }E^\theta = - 0.48\;{\mathrm{V}}\;{\mathrm{vs}}\;{\mathrm{SHE}}$$

3 $${\mathrm{Full}}\;{\mathrm{cell}}:3{\mathrm{I}}^ - + {\mathrm{S}}_4^{2 - } \leftrightarrow {\mathrm{I}}_3^ - + 2{\mathrm{S}}_2^{2 - }E_{\mathrm{cell}} = 1.02\;{\mathrm{V}}$$

I^−^/I_3_^−^ and S^2−^/S_*x*_^2−^ couples are promising redox-active species for high-energy-density flow batteries on account of their high solubility in water and low costs. Aqueous polysulfide/iodide redox flow batteries (SIFBs) have been studied but require further development for practical energy storage^25^. The working principle of the proposed SIFB with polysulfide anolyte and iodide catholyte can be depicted in Eqs. 1–3. For example, Lu et al. reported that an aqueous SIFB delivered an energy efficiency (EE) of ca. 68% at 10 mA cm^−2^ for 70 cycles^25^. However, the poor electrocatalytic activities for the redox reactions of I^−^/I_3_^−^ and S^2−^/S_*x*_^2−^ on carbon material electrode usually restrict the EE and power density of SIFBs. In fact, power density and charge–discharge EE of a practical flow battery are mainly limited by the polarization derived from ohmic resistance, diffusion of active materials, and charge transfer overpotential at electrodes. Furthermore, larger overpotential at electrodes can lead to the evolution of H_2_ and O_2_ in aqueous electrolyte, which results in energy loss and safety problems^3,26^. In general, carbon materials are used as electrodes in most RFBs owing to their high electronic conductivity, good chemical stability, and economical cost^27^. Thus enhancing the electrocatalytic activities of electrodes to facilitate the I^−^/I_3_^−^ and S^2−^/S_*x*_^2−^ redox reactions is an effective way to boost the performances of SIFBs ^27,28^.

Nowadays, regulating electronic structures of active sites via different methods, such as forming solid solution, heteroatom doping, phase controlling, and so on, can effectively improve the catalytic activity by optimized host–guest electronic interactions and the specific adsorption free energy for reactants^29^. From basic semiconductor physics, two opposite space charge regions and a built-in field will be formed when two semiconductors with different energy structure come into contact and reach thermodynamic equilibrium state^30^. The strongly charged region of in this heterojunction would have the potential to modulate the absorption process of reactant species. Furthermore, the built-in field would also contribute to the charge transfer during the catalytic process. Therefore, the design and fabrication of semiconductor junctions would be a feasible strategy to improve the activity of catalysts.

Metal sulfides (e.g., Co, Cu, Pb) with different compositions, structures, or morphologies, including CuS^31,32^, CoS^33–36^, PbS^37,38^, and Cu_2_S/reduced graphene oxide composites^39^, have been shown to have high electrocatalytic activities toward I_3_^−^ and S_*x*_^2−^ redox systems. Cobalt sulfide has various chemical formulas (e.g., CoS_2_, Co_9_S_8_, CoS, Co_3_S_4_, and Co_4_S_3_) and can form heterojunctions with each other due to their different energy structures. The built-in field of a heterojunction (Supplementary Figs. 1 and 2 and Supplementary Table 1) can accelerate the charge carriers and has been explored in photocatalysts, photodetection, photovoltaics, and light-emitting diodes^40–44^. Thus we envisioned that the uneven charge distribution of semiconductor junctions would improve the activity of cobalt sulfide-based electrocatalysts via enhanced charge transfer activities. Herein CoS_2_/CoS n-n heterojunction, with uneven charge distribution, was fabricated and used as positive and negative electrodes to electrochemically catalyze the I^−^/I_3_^−^ and S^2−^/S_*x*_^2−^ redox reactions. The polysulfide/iodide flow battery with the CoS_2_/CoS heterojunction-modified graphite felt (GF) electrodes can deliver a high EE of 84.5% at the current density of 10 mA cm^−2^, a power density of 86.2 mW cm^−2^, and an EE retention of 96% after ca. 1000 h continuous working.

## Results

### Synthesis and characterization

**Fig. 1:**
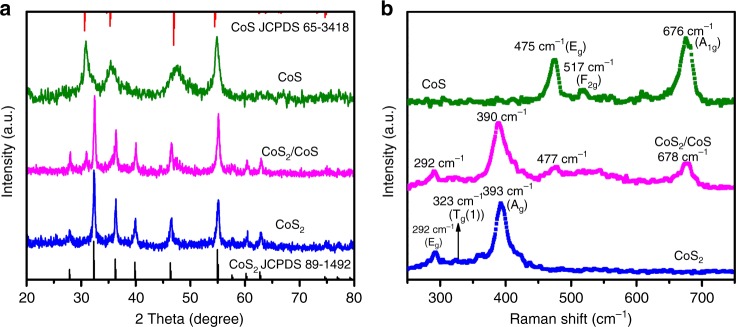
X-ray powder diffraction (XRD) and Raman characterization. **a** XRD patterns of the as-prepared cobalt sulfides compared with the standard patterns of CoS_2_ (JCPDS 89–1492) and CoS (JCPDS 65–3418). **b** Raman spectra of CoS_2_ (blue), CoS_2_/CoS (magenta), and CoS (olive)

CoS_2_, CoS, and CoS_2_/CoS heterojunction were synthesized from CoSO_4_•7H_2_O, urea, and sulfur via tuning the ratio of dimethylformamide (DMF) and ethylene glycol (EG) in a mixed solvent (see “Methods” for details). As shown in Fig. 1a, all diffraction peaks can be indexed well to cubic CoS_2_ (JCPDS 89–1492) and hexagonal CoS (JCPDS 65–3418) when *V*_EG_/*V*_DMF_ = 0.5 and 2, respectively. Notably, mixed phases of cubic CoS_2_ and hexagonal CoS were obtained when *V*_EG_/*V*_DMF_ = 0.9. In Raman spectra, the characteristic peaks are at 292, 323, and 393 cm^−1^, which match well with the reported value of CoS_2_ (Fig. 1b)^45^. The peaks at 475, 517, and 676 cm^−1^ represent the *E*_g_, *F*_2g_, and *A*_1g_ modes of CoS, respectively^46^. Both the characteristic peaks of CoS_2_ (292 cm^−1^, 390 cm^−1^) and CoS (477 cm^−1^, 678 cm^−1^) can be found in the obtained the CoS_2_/CoS heterojunction spectrum. It is noted that the peaks corresponding of CoS_2_ slightly shifts from 393 cm^−1^ of pure CoS_2_ to 390 cm^−1^ of CoS_2_/CoS heterojunction. On the contrary, the peaks corresponding of CoS slightly shifts from 475 cm^−1^ of pure CoS to 477 cm^−1^ of CoS_2_/CoS heterojunction. The opposite shifts of CoS_2_ and CoS diagnostic peaks in the Raman spectra imply an opposite charge transfer due to the formation of a heterojunction.

**Fig. 2:**
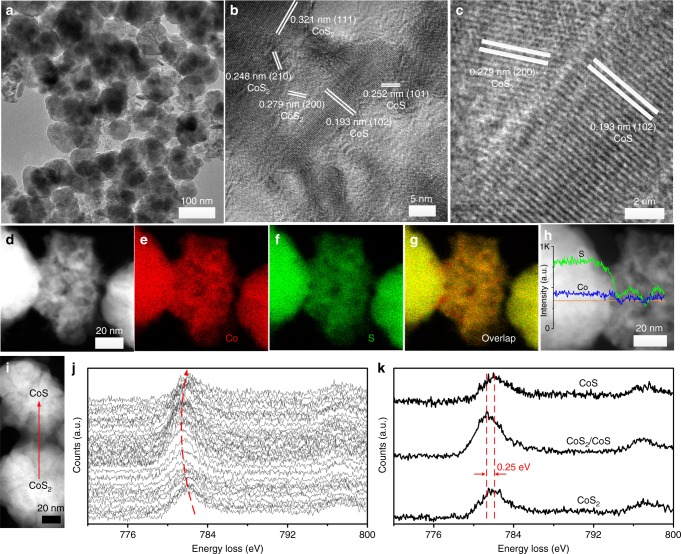
Morphology characterization of CoS_2_/CoS. **a** Transmission electron microscopic (TEM), **b**, **c** high-resolution TEM, **d**, **h** high-angle annular dark-field scanning transmission electron microscopic (HAADF-STEM), **e**–**g** energy-dispersive X-ray spectroscopic (EDX) mapping images, and **h** line-scan EDX of the as-prepared CoS_2_/CoS heterojunction. **i** HAADF-STEM image of CoS_2_/CoS with an arrow indicating electron energy loss spectra (EELs) line-scan direction and **j** corresponding EELs line-scan spectra of the CoS_2_/CoS heterojunction. **k** EELs spectra of Co L_23_ edge selected from center of CoS_2_, heterojunction boundary, and center of CoS, respectively

Transmission electron microscopy (TEM) image (Fig. 2a) shows the CoS_2_/CoS particles of 100 nm in size. Clear lattice fringes and the interface between CoS_2_ and CoS without obvious amorphous area confirmed the formation of heterojunction of CoS_2_/CoS (Fig. 2b, c). As shown in the high-angle annular dark-field scanning transmission electron microscopy (HAADF-STEM) image and elemental mappings in Fig. 2d–g, uniform distributions of Co and S are in accordance with the morphology of the CoS_2_/CoS heterojunction. The line-scan energy-dispersive X-ray spectroscopy (EDX) of the CoS_2_/CoS heterojunction demonstrates that the atomic ratio of S and Co at the left of line-scan spectrum is 2:1 for CoS_2_ and suddenly changes to 1:1 for CoS at the right of line-scan spectrum (Fig. 2h).

To understand the formation of the CoS_2_/CoS heterojunction and the charge distribution at the interface more clearly, line-scan electron energy loss spectra (EELs) of Co L_23_ edge (Fig. 2i–k) in the CoS_2_/CoS heterojunction and X-ray photoelectron spectra (XPS) were further investigated. The chemical shift of Co L_3_ edge of 0.25 eV to lower energy at the heterojunction boundary was observed, indicating the negatively charged characteristics assigned to the side of CoS of the heterojunction. The heterojunction interfaces of other CoS_2_/CoS nanoparticles also have chemical shift evidenced by line-scan EELs spectra (as shown in Supplementary Figs. 3 and 4). However, EELs line scan across a single particle indicated that there is an unchanged chemical state of Co species despite particle thickness (Supplementary Fig. 5). In XPS, sharp peaks of Co 2p and S 2p are detected in all samples (Supplementary Fig. 6), and Co is mainly at a valence state of Co^2+^ in all samples^47^. From the S 2p spectra of pure CoS_2_ and CoS (Supplementary Fig. 7), one can see the typical peaks at 162.3 and 163.5 eV of the bridging S_2_^2−^ in CoS_2_ and the peaks at 161.5 and 162.7 eV of divalent sulfide (S^2−^) in CoS^48–50^. In the CoS_2_/CoS heterojunction, the broad S 2p spectrum can be fitted into two pairs of peaks corresponding to bridging S_2_^2−^ of CoS_2_ (162.4 and 163.5 eV) and divalent sulfide (S^2−^) of CoS (161.3 and 162.7 eV) with a ratio of 1/0.17, which is obtained by integrating peak areas. It is noted that the binding energy of S 2p_3/2_ (S^2−^) in CoS_2_/CoS heterojunction shifts slightly to a lower binding energy by ca. 0.2 eV than that in CoS due to extra negative charges in the region of CoS, while the binding energy of S 2p_3/2_ (S_2_^2−^) is slightly higher than that in CoS_2_^51^. The opposite shift of S 2p_3/2_ demonstrates the distribution of opposite charges in CoS_2_/CoS because of the formation of a heterojunction. The EELs and XPS results confirm the uneven charge distribution at the interface region of the CoS_2_/CoS heterojunction.

### Adsorption behaviors

**Fig. 3:**
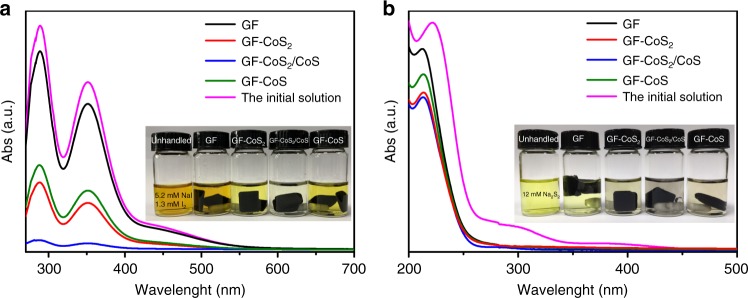
Adsorption characterization of the electrode materials. Ultraviolet-visible spectra of the NaI + I_2_ (**a**) and Na_2_S_2_ (**b**) initial solutions and the solutions after interacting with graphite felt (GF), GF-CoS_2_, GF-CoS_2_/CoS, and GF-CoS, respectively. The loading amount of electrocatalysts onto GF is 1.0 mg cm^−2^. Inset: Visualized adsorption of NaI + I_2_ and Na_2_S_2_ and aqueous solutions on GF, GF-CoS_2_, GF-CoS_2_/CoS, and GF-CoS

The CoS_2_, CoS_2_/CoS, and CoS can be uniformly assembled onto the GF to form an integrated GF-CoS_2_, GF-CoS_2_/CoS, and GF-CoS electrodes, respectively (1.0 mg cm^−2^ loading, Supplementary Figs. 8–10). The adsorption behavior of these electrodes to redox species is a critical factor of the electrocatalytic process. As shown in Fig. 3a, the NaI_3_ aqueous solution with GF-CoS_2_/CoS exhibits more obvious decoloration than ones with the GF, GF-CoS_2_, and GF-CoS and nearly turns to a colorless solution. In addition, the ultraviolet–visible (UV-Vis) absorption of the NaI_3_ solution after adsorption with GF-CoS_2_/CoS, the typical absorption peaks of I_3_^−^ ions are nearly disappeared. The sharp contrasts indicate that CoS_2_/CoS heterojunction has remarkable adsorption effect on iodide species. The adsorption capacity of the materials for polysulfides has also been investigated (Fig. 3b) and shows a similar sequence to their absorptivity to iodide solutions.

To qualitatively elucidate the interactions between the (CoS_2_ (200), CoS (100) and the heterostructure CoS_2_ (200)/CoS (100) with iodide or polysulfide ions, density functional theory (DFT) was used to calculate their adsorption energy (Table 1, Supplementary Figs. 11 and 12). The DFT results revealed that the adsorption energies of the CoS_2_ (200)/CoS (100) heterojunction to polysulfide or iodide ions is higher than the individual pure structures of CoS_2_ (200) and CoS (100) surface, which is consistent with the experimental results. Constructing heterojunction improves the adsorption energy of the system and the negative value of adsorption energy indicates that these adsorption processes are thermodynamically favorable. The above results suggest the significantly promoted affinity of CoS_2_/CoS heterojunction with polysulfide and iodide molecules in comparison to CoS_2_ or CoS.

**Table 1:** Surface calculation data of CoS_2_, CoS, and CoS_2_/CoS

Materials	Typical surface	Space group	Lattice parameters	*E*_ad_ (Polysulfide)/eV				*E*_ad_ (Iodide)/eV		
				S_6_^2−^	S_4_^2−^	S_2_^2−^	S^2−^	I_3_^−^	I_2_	I
CoS_**2**_	(200)	Pa-3 (205)	a = b = c = 5.506	−0.32	−0.24	−0.16	−0.11	−1.47	−0.18	−0.10
CoS	(102)	P63/mmc (194)	a = b = 3.347, c = 5.139	−0.28	−0.19	−0.11	−0.07	−1.23	−0.13	−0.07
CoS_**2**_/CoS heterojunction	CoS_2_ (200)/CoS (102)	—	—	−0.63	−0.57	−0.34	−0.22	−2.13	−0.44	−0.22

### Electrocatalytic activity investigation

**Fig. 4:**
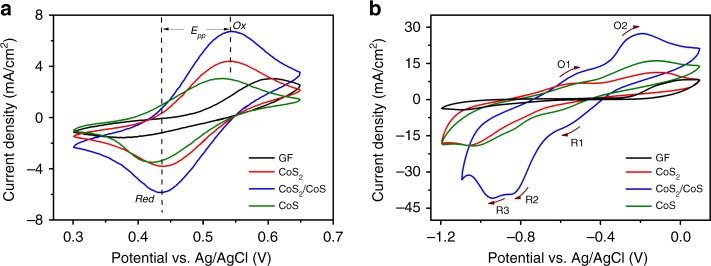
Cyclic voltammetry characterization. The cyclic voltammograms (CVs) of the catalysts deposited on glassy carbon in 4 mM NaI + 0.5 M NaCl solution (**a**) and 0.06 M Na_2_S + 0.02 M S + 0.5 M NaCl solution (**b**) at a scan rate of 50 mV s^−1^

The effect of the CoS_2_/CoS heterojunction on the electrocatalytic activity of I^−^/I_3_^−^ and S^2−^/S_*x*_^2−^ redox reactions were studied by cyclic voltammogram (CV). In Fig. 4a, each curve exhibits a pair of peaks corresponding to the redox reactions of I^−^/I_3_^−^ couples using GF, CoS_2_, CoS, and CoS_2_/CoS electrodes. The peak-to-peak separation (*E*_pp_) and peak current density are vital parameter indicators to evaluate the electrocatalytic property of electrodes^52^. The corresponding parameters are listed in Table 2. Compared with CoS_2_ and CoS, the smallest *E*_pp_ (0.10 V) and highest peak current density of CoS_2_/CoS indicate that the heterojunction is more effective to facilitate the redox reaction of I^−^/I_3_^−^. The 1.15 value of *J*_Ox_/|*J*_Red_| for CoS_2_/CoS also implies its better reversibility to I^−^/I_3_^−^ redox reaction. Two typical pairs of redox peaks of electrodes in Supplementary Fig. 13 can be ascribed to the redox reaction of I^−^/I_3_^−^ and I_3_^−^/I_5_^−^. The linear relationship between peak currents and the square root of scan rate (*ν*^1/2^, Supplementary Figs. 14 and 15) indicates the diffusion-controlled process of the I^−^/I_3_^−^ redox reaction. The diffusion coefficient *D* of I^−^ can be estimated by the Berzins–Delahay equation (Supplementary Table 2), which follows an order of GF < CoS < CoS_2_ < CoS_2_/CoS, being consistent with the order of the electrode catalytic performances. Moreover, the 200 consecutive CVs indicates the better stability of CoS_2_/CoS than CoS_2_ and CoS for the I^−^/I_3_^−^ redox reaction (Supplementary Fig. 16). As shown in Fig. 4b, the CoS_2_/CoS electrode indeed greatly enhanced the electrochemical reactivity of the polysulfide couple and resulted in resolved multiple redox peaks of polysulfides, which are integrated into one pair of asymmetry broad peaks in case of other electrodes. The multiple electron transfers are present in the potential range of ~−1.2 V–0.1 V vs Ag/AgCl, which has the two oxidation peaks and three reduction peaks. This suggests that the polysulfide may undergo more complicated electrochemical reactions with likely involvement of more polysulfide species, e.g., S_*x*_^2−^ (*x* = from 2 to 8), which has been demonstrated in both aqueous and nonaqueous electrolytes^53,54^.

**Table 2:** Parameters obtained from CV curves for I^−^/I_3_^−^ on GF, CoS_2_, CoS_2_/CoS, and CoS, respectively

Samples	*J*_Ox_ [mA cm^−2^]	*J*_Red_ [mA cm^−2^]	*J*_Ox_/*|J*_Red_*|*	*E*_Ox_ [V]	*E*_Red_ [V]	*E*_pp_ [V]
GF	3.02	−1.58	1.91	0.61	0.38	0.23
CoS_2_	4.38	−3.79	1.16	0.54	0.44	0.10
CoS_2_/CoS	6.72	−5.85	1.15	0.54	0.44	0.10
CoS	3.04	−3.48	0.87	0.53	0.42	0.11

*CV* cyclic voltammogram, *J*_*Red*_ the cathodic peak current density, *J*_*Ox*_ the anodic peak current density, *E*_*pp*_ peak-to-peak separation

### Flow battery studies

**Fig. 5:**
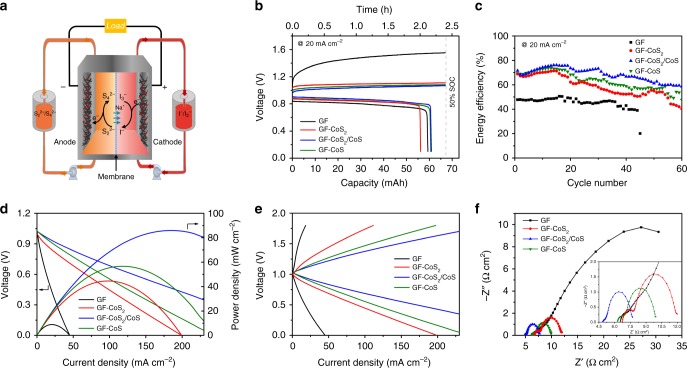
Configuration and performance of the polysulfide/iodide redox flow batteries (SIFBs). **a** Scheme of the proposed SIFB cell configuration. **b** The first charge and discharge curves of SIFBs based on graphite felt (GF), GF-CoS_2_, GF-CoS_2_/CoS, and GF-CoS electrodes with N115 and N117 as the separator at 20 mA cm^−2^ with the 50% state of charge (SOC). **c** The galvanostatic cycling energy efficiency of the SIFBs at 20 mA cm^−2^. **d** The discharge polarization and power density curves of the SIFBs at 50% SOC. **e** The polarization curves of both charge and discharge with the same flow cell at 50% SOC. **f** The electrochemical impedance spectroscopic (EIS) plots of SIFBs with GF, GF-CoS_2_, GF-CoS_2_/CoS, and GF-CoS electrodes in the frequency range of 100 kHz to 100 mHz with 5 mV ac oscillation

The schematic representation for the proposed SIFB cell configuration is shown in Fig. 5a. SIFB cells based on GF, GF-CoS_2_, GF-CoS_2_/CoS, and GF-CoS as both positive and negative electrodes were assembled. A combination of N115 and N117 were used as the two-layer separator to promote the Na^+^ cation transport and limit the crossover of active species^24^. For battery tests, the electrolyte volume was 5 mL for both 2.0 M NaI + 0.5 M I_2_ catholyte and 2.0 M Na_2_S_2_ anolyte and the flow rate was fixed at 10 mL min^−1^. Figure 5b, c and Supplementary Fig. 17 show the galvanostatic cycling performance of the polysulfide/iodide flow battery at 20 mA cm^−2^ with the charge state of 50% state of charge (SOC). The corresponding charge/discharge voltage profiles of SIFBs are plotted in Supplementary Fig. 18. The GF-CoS_2_/CoS displayed the highest EE value than others electrodes at the same charge/discharge current density resulting from the reduced charge/discharge overpotentials. The voltage efficiency (VE) value is a derivative of the coulombic efficiency (CE) and EE (VE = EE/CE). The SIFB with GF-CoS_2_/CoS delivered an EE of 71.6% at 20 mA cm^−2^ and 84.0% capacity retention for 60 cycles, which is significantly higher than the one with bare GF electrodes (47.8% EE). The SIFBs with GF-CoS_2_ and GF-CoS delivered an EE of 68.9% and 69.4% at 20 mA cm^−2^ and 58.5% and 68.6% capacity retention for 60 cycles, respectively. Furthermore, the trend of CE, VE, and EE over current densities of SIFBs are outlined in Supplementary Figs. 19 and 20. CE stayed above ca. 91% at all current densities. The EE of GF-CoS_2_/CoS decreased from 72.0 % for 15 mA cm^−2^ to 55.9% for 50 mA cm^−2^. The observed trends for the VE and EE typically attribute to the increased cell overpotential at higher current densities. In addition, GF-CoS_2_/CoS electrode has the highest EE of 84.5% at 10 mA cm^−2^, and the EE retention was 96% after 500 cycles of continuous working at 10% SOC (ca. 1000 h) with multiple electrolyte refreshments (Supplementary Fig. 21). As shown in Fig. 5d, the discharge polarization curves and power density curves of SIFBs clearly reveal that the GF-CoS_2_/CoS electrode has the highest power density of 86.2 mW cm^−2^ compared to GF (8.9 mW cm^−2^), GF-CoS_2_ (44.6 mW cm^−2^), and GF-CoS (56.6 mW cm^−2^), respectively. The charge and discharge polarization curves of SIFBs shows that the open circuit voltage of the GF-CoS_2_/CoS cell is 1.03 V, which is in good agreement with the theoretical value of 1.02 V. And the hysteresis voltage between charge and discharge processes of the SIFB cell using GF-CoS_2_/CoS electrodes is smaller than other electrodes under the same current density (Fig. 5e). Electrochemical impedance spectroscopy (EIS) was used to investigate the charge transfer resistance (*R*_ct_) and evaluate the electrode activity. The EIS plots of the SIFBs with different electrodes are simulated (Fig. 5f) and the results are summarized in Table 3. In the Nyquist plot of electrodes, a semicircle is observed, which corresponds to *R*_ct_ at the electrolyte/electrode interface^55^. It is clearly seen that the *R*_ct1_s are 3.66, 1.03, 0.46, and 0.84 Ω cm^2^, respectively, for GF, GF-CoS_2_, GF-CoS_2_/CoS, and GF-CoS electrodes. The *R*_ct2_s are 36.6, 4.19, 2.40, and 2.83 Ω cm^2^, respectively, for GF, GF-CoS_2_, GF-CoS_2_/CoS, and GF-CoS electrodes. These results further illustrate that the CoS_2_/CoS heterojunction can facilitate the charge transfer process and improve diffusion dynamics, which fits well with the CV experiments. To evaluate the stability of electrodes, the catholyte of SIFBs based on the GF-CoS_2_ GF-CoS_2_/CoS, and GF-CoS electrodes were sampled after 60 cycles and further tested by the inductively coupled plasma spectroscopy (ICP). The ICP results showed that no cobalt was detected in the catholyte of GF-CoS_2_/CoS and GF-CoS electrodes and 0.3 ppm cobalt was detected using GF-CoS_2_ electrode, which confirms the good stability of GF-CoS_2_/CoS electrode and no particles were fallen off. Supplementary Fig. 22 exhibits that the surface morphology of the GF-CoS_2_/CoS electrode after 60 cycles at a current density of 20 mA cm^−2^ still maintained its integrity similar to the fresh one. All of the results discussed above demonstrate remarkable power densities and promising long-term cycling stability of the GF-CoS_2_/CoS electrode.

**Table 3:** Summary of simulation results from EIS spectra of GF, GF-CoS_2_, GF-CoS_2_/CoS, and GF-CoS electrodes, respectively

Electrode	*R*_s_ [Ω cm^2^]	*R*_ct1_ [Ω cm^2^]	*R*_ct2_ [Ω cm^2^]
GF	6.39	3.66	36.6
GF-CoS_2_	6.74	1.03	4.19
GF-CoS_2_/CoS	4.84	0.46	2.40
GF-CoS	6.26	0.84	2.83

*EIS* electrochemical impedance spectroscopy, *GF* graphite felt

## Discussion

In summary, the CoS_2_/CoS n-n heterojunction with uneven charge distribution was prepared by a rationally designed solvothermal system via simply adjusting the proportion of DMF and EG in the mixed solvent and employed as highly active electrocatalysts in aqueous SIFBs. The charged surfaces derived from space charge regions of the CoS_2_/CoS heterojunction can enhance the electrochemical activities of I^−^/I_3_^−^ and S^2−^/S_*x*_^2−^ redox reactions by the improved absorptivity to charged ions and promoted the charge transfer process. Therefore, the flow cell with the GF-CoS_2_/CoS electrode gives a high EE of 84.5% at 10 mA cm^−2^ and the EE retention of 96% after ca. 1000 h continuous working. Moreover, the GF-CoS_2_/CoS electrode has a much higher power density of 86.2 mW cm^−2^ compared to GF electrode (8.9 mW cm^−2^). The design of heterojunction electrocatalysts would offer an effective strategy to enhance the performance and competitiveness of SIFBs and other RFBs.

## Methods

### Materials

Sodium iodide (NaI, 99%), sodium sulfide nonahydrate (Na_2_S∙9H_2_O, >98%), sodium chloride, sublimed sulfur (99.5%), sulfuric acid (H_2_SO_4_, 98%), hydrogen peroxide (H_2_O_2_, 30 wt% in H_2_O), CoSO_4_∙7H_2_O, urea, DMF, and EG were purchased from Sinopharm Chemical Reagent Co., Ltd. Iodine (I_2_, 99.8%) was purchased from Shanghai Titan Scientific Co., Ltd. Nafion membrane (N115 and N117, Dupont, DE, USA) was received from Innochem. Sodium polysulfide (Na_2_S_*x*_) electrolytes were prepared by mixing stoichiometric ratios of Na_2_S and S at room temperature. Sodium triiodide (NaI_3_) electrolytes were prepared by mixing stoichiometric ratios of NaI and I_2_ at room temperature.

### Synthesis of the electrode materials

Prior to synthesis, the as-received GF (Beijing Jinglong Carbon Technology Co., Ltd.) was first soaked overnight in 6 M H_2_SO_4_. It was rinsed with deionized (DI) water until the effluent pH is near 7 and then heat treated at 300 °C for 2 h. The CoS_2_/CoS heterojunction was synthesized by a facile and one-step hydrothermal method. In a typical procedure, 2 mmol CoSO_4_∙7H_2_O, 10 mmol urea, and 25 mmol sublimed sulfur were mixed in 70 mL mixed solution of EG and DMF (*V*_EG_/*V*_DMF_ = 0.9). The precursor solution was magnetically stirred for 1 h and subsequently added into a 100 mL Teflon-lined autoclave. The autoclave was kept at 180 °C for 12 h in an oven. After cooling down, the product was collected by centrifugation, washed with DI water and absolute ethanol several times, and dried at 60 °C for 12 h under vacuum. Similarly, to obtain the GF-CoS_2_/CoS electrode, the GF (1.3 cm × 1.3 cm × 3 mm) was placed inside the precursor solution and the reaction temperature was kept at 180 °C for 12 h. After cooling down, the GF loaded with CoS_2_/CoS heterojunction was washed in an ultrasonic cleaner by water several times until no obvious CoS_2_/CoS particles were observed in the wastewater stream and finally dried at 60 °C for 12 h under vacuum. For comparison, the pure CoS_2_ and CoS were also synthesized using a similar process via tuning the mixed solvent of DMF and EG. When V_EG_/*V*_DMF_ = 0.5 and 2, the products were CoS_2_ nanospheres and CoS, respectively. Similarly, in the GF-CoS_2_ and GF-CoS electrode experiments, the GFs were inserted into the corresponding precursor solution and the reaction temperature was kept at 180 °C for 12 h. Finally, the GF-CoS_2_ and GF-CoS electrodes were collected. Their mass loadings on GF were calculated through the weight change of the GF. The mass loadings of CoS_2_, CoS_2_/CoS, and CoS on the GF were 1.07, 0.83, and 1.12 mg cm^−2^, respectively.

### Assembly of aqueous SIFBs

The Supplementary Fig. 23 showed the configurations of aqueous SIFB cells. The flow cell used in this study was similar to previous literature reports with interdigitated flow fields and a geometric active area of 1.69 cm^2^ (1.3 × 1.3 cm^2^)^1^. Carbon papers (Beijing Jinglong Carbon Technology Co., Ltd.) were used as the current collector. The GF, GF-CoS_2_, GF-CoS_2_/CoS, and GF-CoS electrodes were used as both positive and negative electrodes. Commercially available Nafion membranes (N115 and N117) were utilized as separators. First, membranes were treated with 5% H_2_O_2_ under 80 °C for 1 h and then were transferred to 5% H_2_SO_4_ at 80 °C for 1 h. Finally, 1 M NaOH aqueous solution was used to change the H-type (i.e., proton conductive) Nafion membranes to Na-type (i.e., Na^+^ conductive) under 80 °C for 2 h. The membranes were rinsed in DI water for 30 min to wash away the chemicals after each step. Electrolytes were prepared at room temperature under continuously bubbling argon gas for 30 min to prevent oxidation by oxygen before the use. Two half cell bodies with the GF, GF-CoS_2_, GF-CoS_2_/CoS, or GF-CoS electrodes as both positive and negative electrodes and the Nafion membrane (N115 and N117) in between were assembled in the ambient air. A peristaltic pump (BT100LC, Baoding Chuang Rui Precision Pump Co., Ltd) was used to drive electrolytes through the flow cell and reservoirs. Silicone tube (1.6 mm inner diameter) was used to circulate the electrolyte through the system.

### Characterization

The as-prepared products were characterized on an XRD (Bruker-D8 advance) equipped with a Cu Kα radiation source (*λ* = 1.5418 Å) at a scanning rate of 6 ° min^−1^; X-ray tube voltage and current were set at 40 kV and 40 mA, respectively. SEM, TEM, and HAADF-STEM images were taken with an FEI Nova NanoSEM NPE218, JEM-2100, and JEM-ARM200F, respectively. The samples were prepared by dropping ethanol dispersion of samples onto carbon-coated copper TEM grids using pipettes and dried under ambient condition. EELs were recorded using an FEI Talos F200X equipped with super-EDX and energy filter (Gatan GIF Quantum ER 965) operated at 200 kV under STEM mode. By using Gatan Quantum 965 with dual EELs capability, both low- and high-loss region were collected near simultaneously, which allows accurate measurement of chemical shifts as EELs SI analysis was carried out. XPS was performed on an AXIS ULTRA DLD X-ray photoelectron spectrometer. UV-Vis absorption spectrum was obtained from Lambda 750S (Perkin Elmer, Inc., USA). Elemental content was tested by EDX and ICP-optical emission spectroscopy (iCAP7600). Raman spectra were recorded by a DXR Raman spectrophotometer (Thermo Fisher Scientific) at an excitation radiation wavelength of 532 nm.

### Visualized adsorption test

The GF, GF-CoS_2_, GF-CoS_2_/CoS, and GF-CoS with the same mass were added into 10 mL of iodide (5.2 mM NaI + 1.3 mM I_2_) or polysulfide (12 mM Na_2_S_2_) aqueous solution separately, and the mixtures were vigorously stirred 12 h to realize through adsorption. The UV-Vis spectroscopy of the solutions was further recorded for comparison.

### Electrochemical measurements

CV tests were performed in a three-electrode configuration with a Zahner Zennium CIMPS-1 electrochemical workstation. For fabrication of the working electrodes, 10 mg of catalysts (for example, CoS_2_/CoS) was dispersed in 500 μL of DI water, 500 μL of isopropanol, and 20 μL of 5 wt% Nafion solution to form a homogeneous ink. Ten microliters of the catalyst ink was loaded onto a glassy carbon electrode of 3 mm in diameter. Then the electrode was dried at room temperature. The electrochemical studies were recorded with a three-electrode system containing an aqueous solution of 4 mM NaI + 0.5 M NaCl or 0.06 M Na_2_S + 0.02 M S + 0.5 M NaCl at a scan rate of 50 mV s^−1^ at room temperature, in which Pt sheet and Ag/AgCl electrode worked as the counter electrode and the reference electrode, respectively. The Mott–Schottky plot was characterized in a three-electrode system containing a 0.5 mol L^−1^ Na_2_SO_4_ solution, in which Pt worked as the counter electrode, the samples as the working electrode, and Ag/AgCl as the reference electrode, respectively.

### Flow cell tests

The galvanostatic characterizations of the SIFB cells were conducted on battery testing system (LAND, CT2001A, Wuhan LAND electronics Co., Ltd). The electrolyte volume was 5 mL for both 2.0 M NaI + 0.5 M I_2_ catholyte and 2.0 M Na_2_S_2_ anolyte and the electrolyte flow rate was fixed at 10 mL min^−1^ throughout all the experiments. The theoretical capacity was calculated by catholyte (the iodide part), which was 5 mL of 2.0 M NaI + 0.5 M I_2_, with a nominal capacity of 134 mAh. The charge process is limited to the capacity-limiting factor for the full cell. The flow cell was galvanostatically charged to 50% SOC (capacity cutoff) and discharge to 0.1 V voltage cutoff. The flow cell was operated at current densities from 15 to 50 mA cm^−2^. During the cycle test, the electrolytes are filled with N_2_. The extended cycling experiment was conducted at 20 mA cm^−2^. Using the Zahner Zennium CIMPS-1 electrochemical workstation, at the charged state, the EIS measurement was conceived in the frequency range of 100 kHz to 100 MHz with 5 mV ac oscillation.

### Computational methods

In order to optimize both CoS_2_ and CoS structures, the exchange correlation function, Perdew, Burke, and Ernzerhof of the generalized gradient approximation with Koelling–Hamon relativistic treatment and spin polarization assumption is employed. Broyden–Fletcher–Goldfarb–Shanno geometry optimization is used for cell optimization (at fixed internal stress). The interaction between valence electrons and the ionic core is described by using On-The-Fly-Generation ultra soft pseudo potential. The kinetic cutoff energy for convergence test is 300 eV, a *k*-point set mesh (3 × 3 × 1) parameter is used for Brillouin zone sampling^56,57^. The threshold for self-consistent field iterations used is 2.0 × 10^−6^ eV atom^−1^. The convergence tolerance parameters of the optimized calculation are the tolerance for energy 2.0 × 10^−5^ eV atom^−1^, the maximum force of 0.05 eV Å^−1^, and maximum displacement of 2 × 10^−3^ Å^58,59^.

## Supplementary information

Supplementary Info

## Data Availability

The data underlying Figs. 1, 2a–g, and 6d, 6f and Supplementary Figs. 3–5 and 21 are provided as a Source Data file. The other data that support the findings of this study are available from the corresponding author on reasonable request.
