# Supplementary material for: Highly active nanostructured CoS2/CoS heterojunction electrocatalysts for aqueous polysulfide/iodide redox flow batteries
Source: Nat Commun. 2019 Jul 29;10:3367. doi: 10.1038/s41467-019-11176-y (PMC6662769; doi:10.1038/s41467-019-11176-y)
Supplement: Supplementary file 1 — Supplementary Info [file 41467_2019_11176_MOESM1_ESM.doc]

Highly Active Nanostructured CoS_2_/CoS Heterojunction Electrocatalysts for Aqueous Polysulfide/Iodide Redox Flow Batteries

Ma et al.


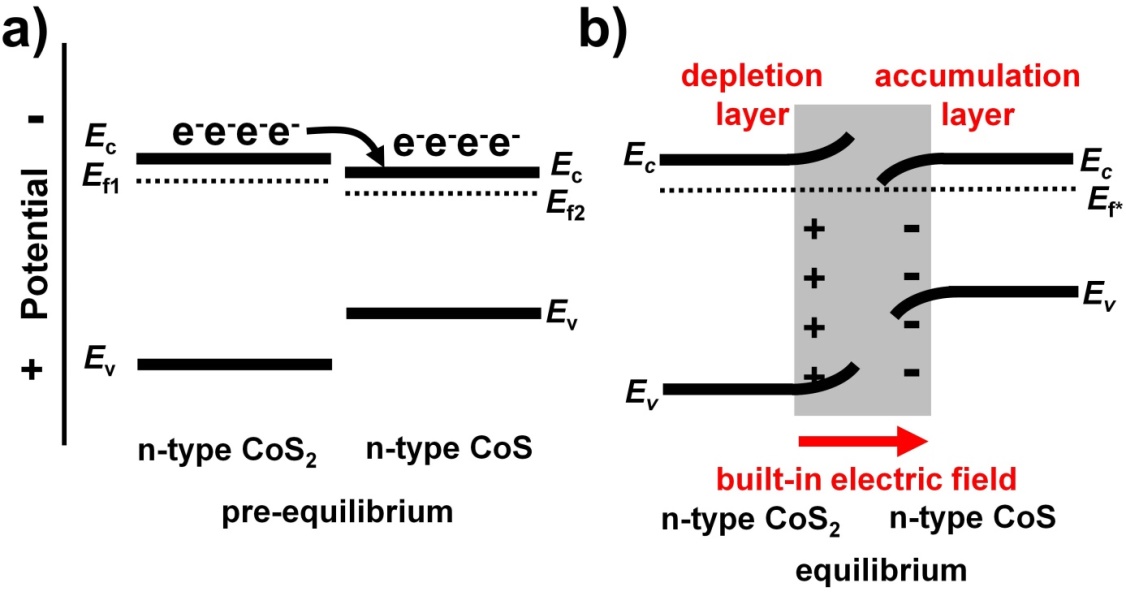


**Supplementary Fig. 1** Schematic diagrams of the band structure of CoS_2_/CoS n-n heterojunction. (**a**) per-equilibrium and (**b**) equilibrium states of CoS_2_/CoS n-n heterojunction


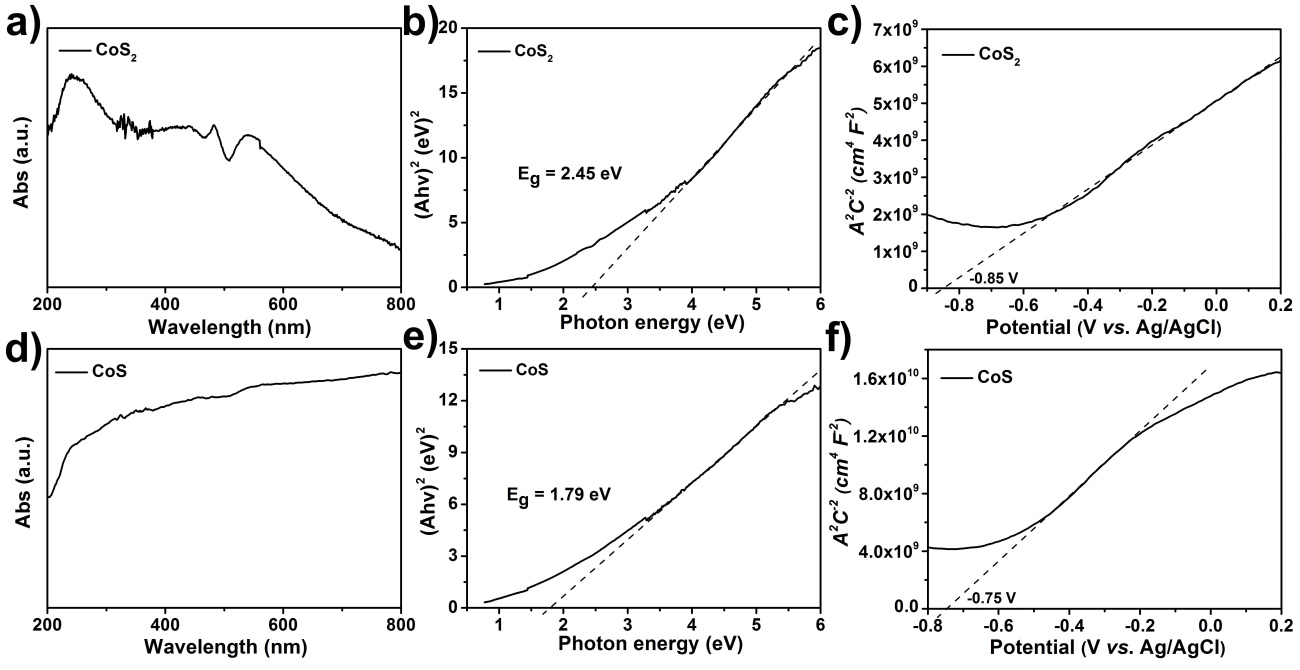


**Supplementary Fig. 2** UV-Vis and Mott-Schottky characterization. UV-Vis spectra, the plots of (*Ahv*)^2^ versus *hv*, the Mott-Schottky plots at the frequency of 3000 Hz for as-prepared CoS_2_ (**a**, **b**, **c**) and CoS (**d**, **e**, **f**).

**Supplementary Table 1.** Optical properties of CoS_2_ and CoS.

| Sample | *E_fb_* (V) | *E_g_* (eV) | *E_CB_* (eV) | *E_VB_* (eV) |
| --- | --- | --- | --- | --- |
| CoS_2_ | -0.65 | 2.45 | -0.85 | 1.60 |
| CoS | -0.55 | 1.79 | -0.75 | 1.04 |

**Supplementary Note 1**

The optical band gap (*E_g_*), valence band (*E_VB_*), and conductive band (*E_CB_*) levels of samples can be calculated (Supplementary Fig. 2) and the related results were summarized in Supplementary Table 1. Using the Boltzmann distribution to describe the distribution of electrons in the space charge region and Gauss’law relating the electric field through the interface to the charge contained within that region, Poisson’s equation can be solved to give the Mott-Schottky equation S1:


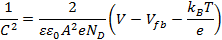
 (Equation S1)

Here *C* and *A* are the interfacial capacitance and area, respectively, *N_D_* the number of donors, *V* the applied voltage, *k_B_* is Boltzmann’s constant, *T* the absolute temperature, and *e* is the electronic charge. Therefore, a plot of 1/*C*^2^ against *V* should yield a straight line from which *V_fb_* can be determined from the intercept on the *V* axis.^1^ As shown in Supplementary Fig. 2, the Mott-Schottky plots of CoS_2_ and CoS exhibit positive slopes. This is a typical n type semiconductors.^2^ The flat band potentials (*E_fb_*) derived from the *x*-intercept in the Mott-Schottky plots of CoS_2_ and CoS are -0.85 V and -0.75 V *versus* Ag/AgCl, which are equal to -0.65 V and -0.55 V *versus* the normal hydrogen electrode (NHE), respectively. It is generally believed for many n-type semiconductors that *E_fb_* is about 0.1-0.3 V more negative than their conduction band position (*E_CB_*) and it is usually defined as -0.20 eV.^3^ Thereby, the *E_CB_* position for CoS_2_ and CoS can be approximately estimated as -0.85 and -0.75 eV, and their valence band potential (*E_VB_*) are correspondingly determined to be 1.60 and 1.04 eV, respectively.


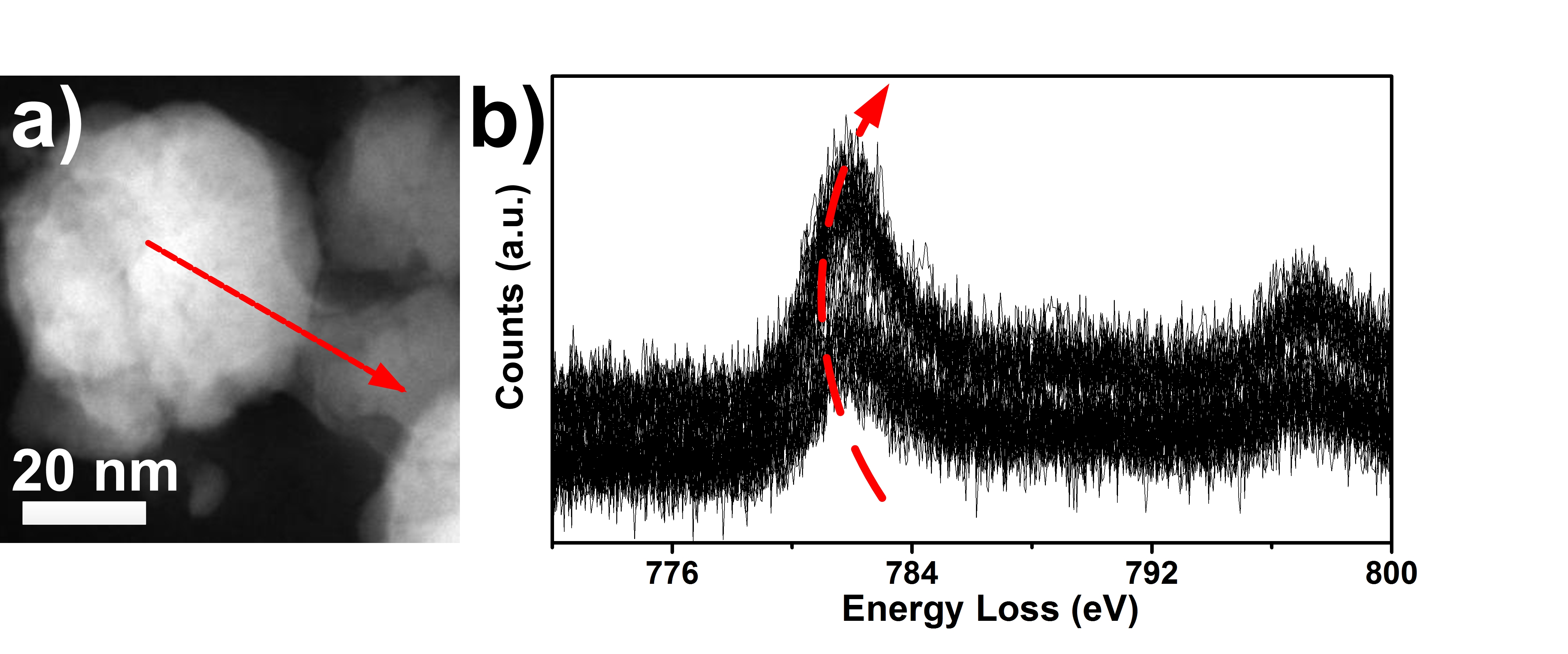


**Supplementary Fig. 3** HAADF-STEM and Electron Energy loss spectra. (**a**) HAADF-STEM image of CoS_2_/CoS with an arrow indicating EELs line-scan direction and corresponding EELs line-scan spectra of the CoS_2_/CoS heterojunction (**b**).


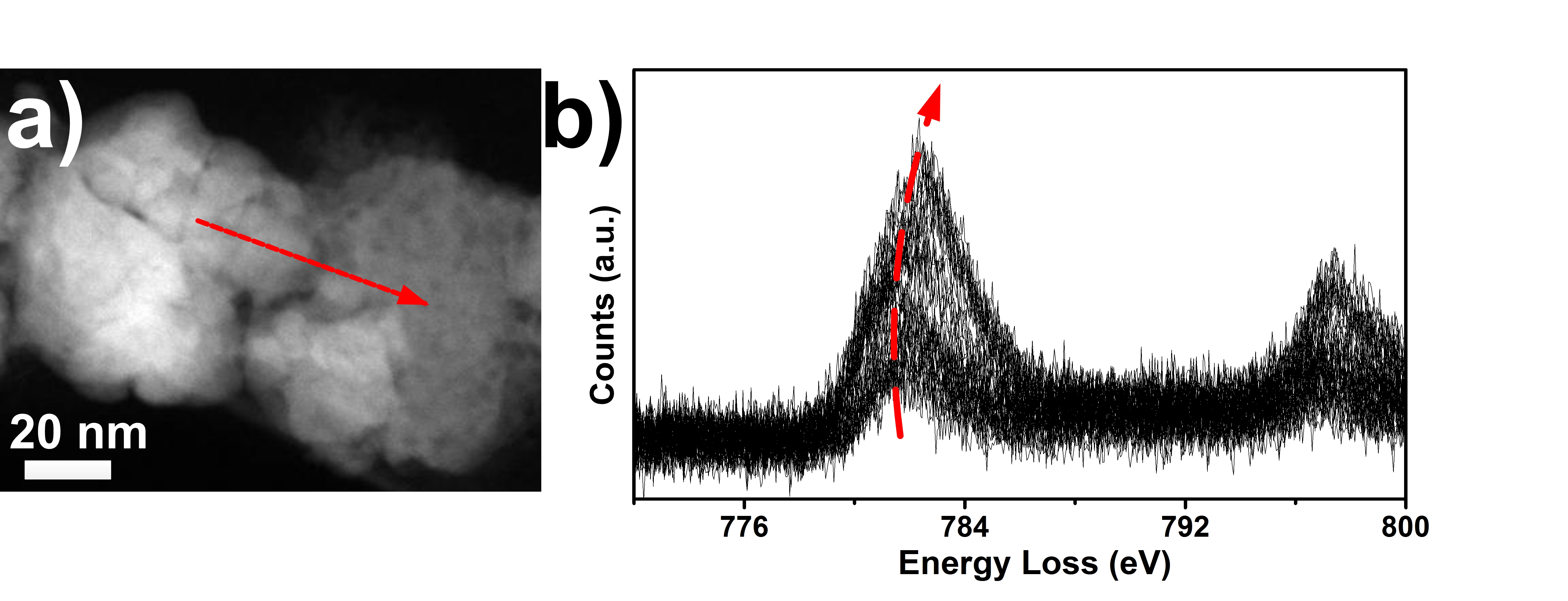


**Supplementary Fig. 4** HAADF-STEM and Electron Energy loss spectra. (**a**) HAADF-STEM image of CoS_2_/CoS with an arrow indicating EELs line-scan direction and corresponding EELs line-scan spectra of the CoS_2_/CoS heterojunction (**b**).


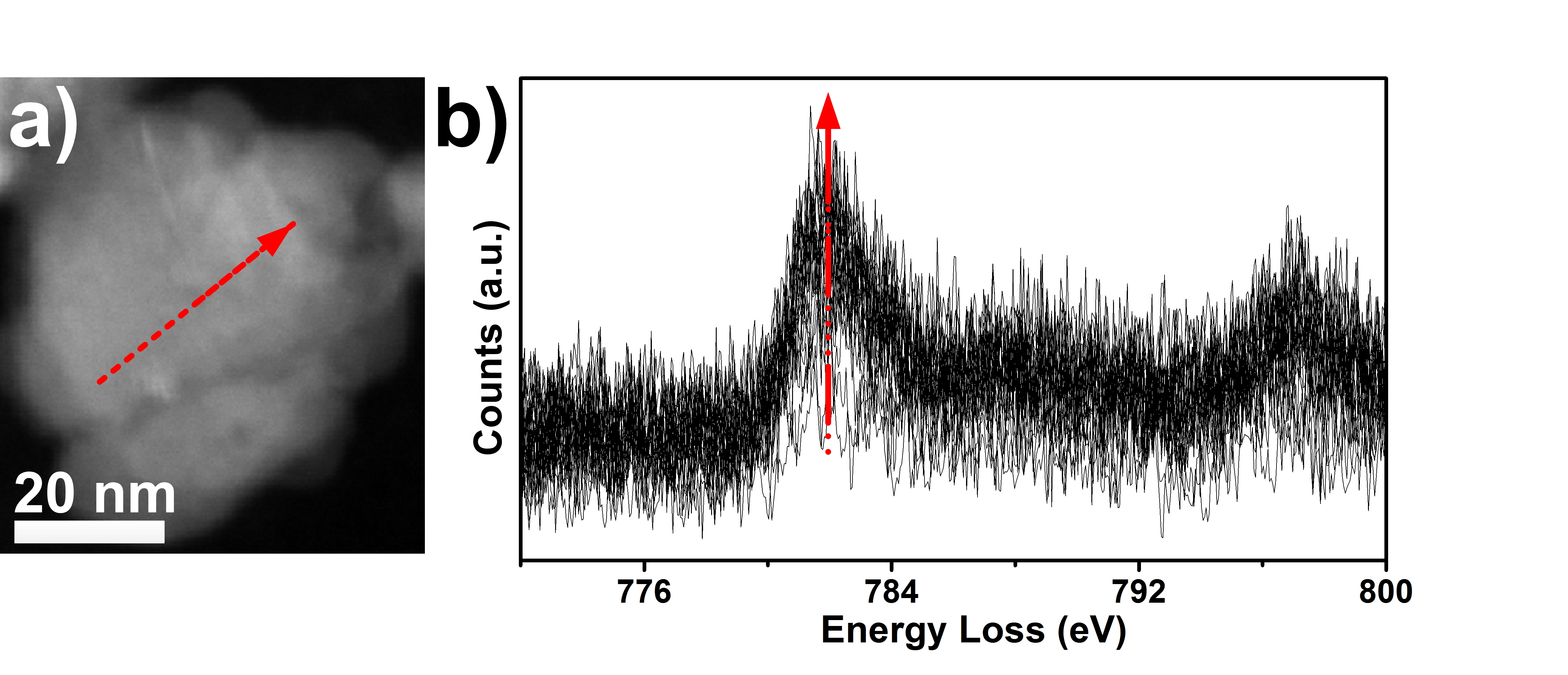


**Supplementary Fig. 5** HAADF-STEM and Electron Energy loss spectra. (**a**) HAADF-STEM image of pure CoS nanoparticle with an arrow indicating EELs line-scan direction and corresponding EELs line-scan spectra of the pure CoS nanoparticle (**b**).


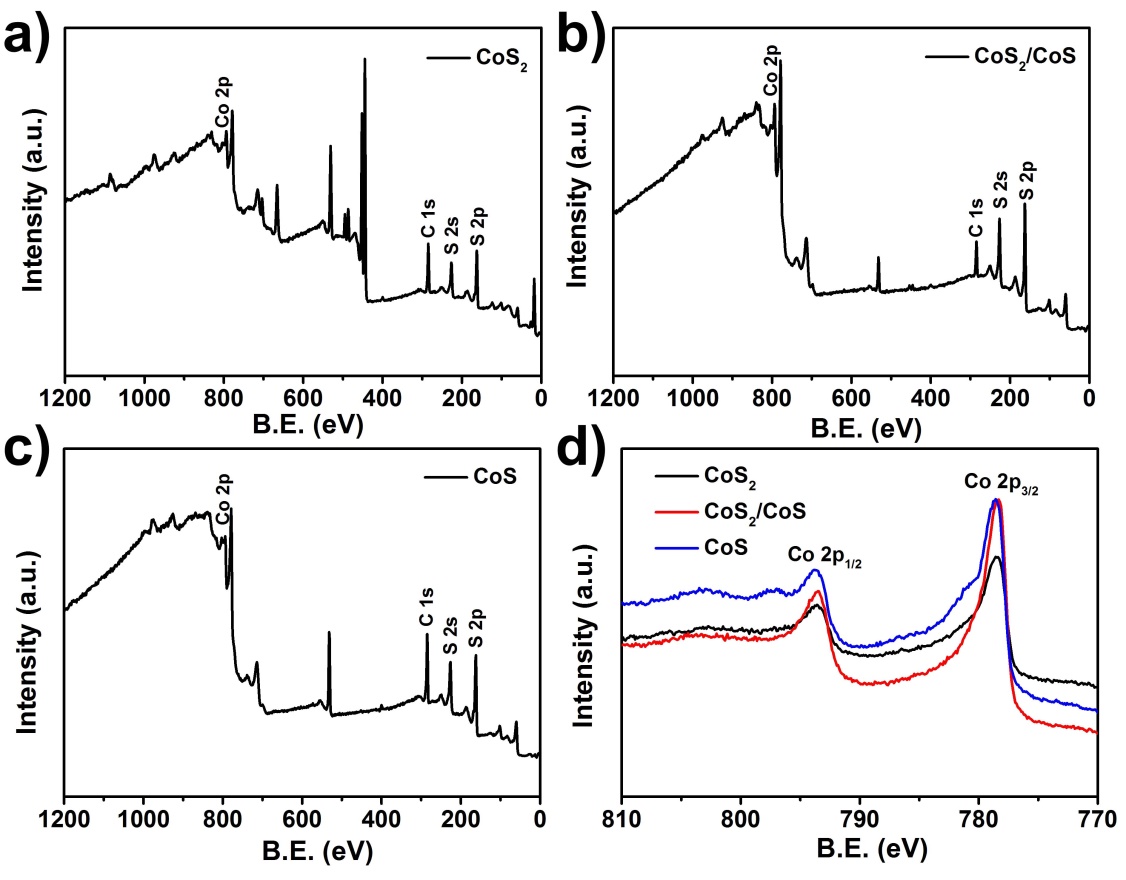


**Supplementary Fig. 6** XPS characterization. XPS survey spectra of (**a**) CoS_2_, (**b**) CoS_2_/CoS and (**c**) CoS. (**d**) High-resolution Co 2p XPS spectra of CoS_2_, CoS_2_/CoS and CoS.


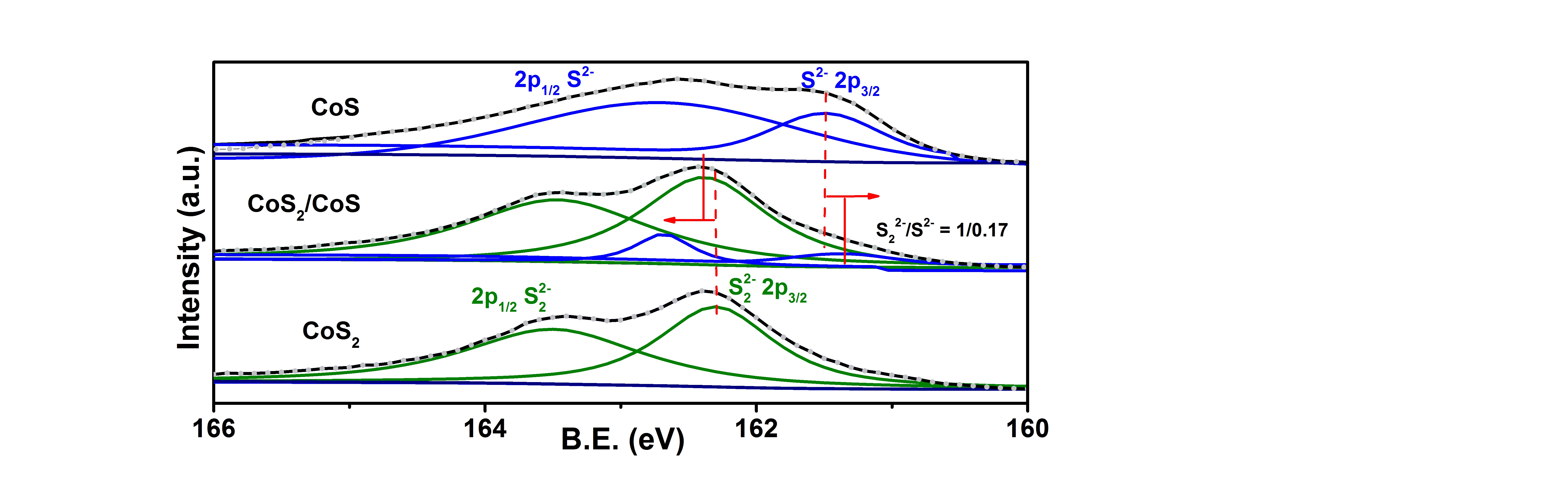


**Supplementary Fig. 7** XPS characterization. High-resolution S 2p XPS spectra of CoS_2_, CoS_2_/CoS and CoS.


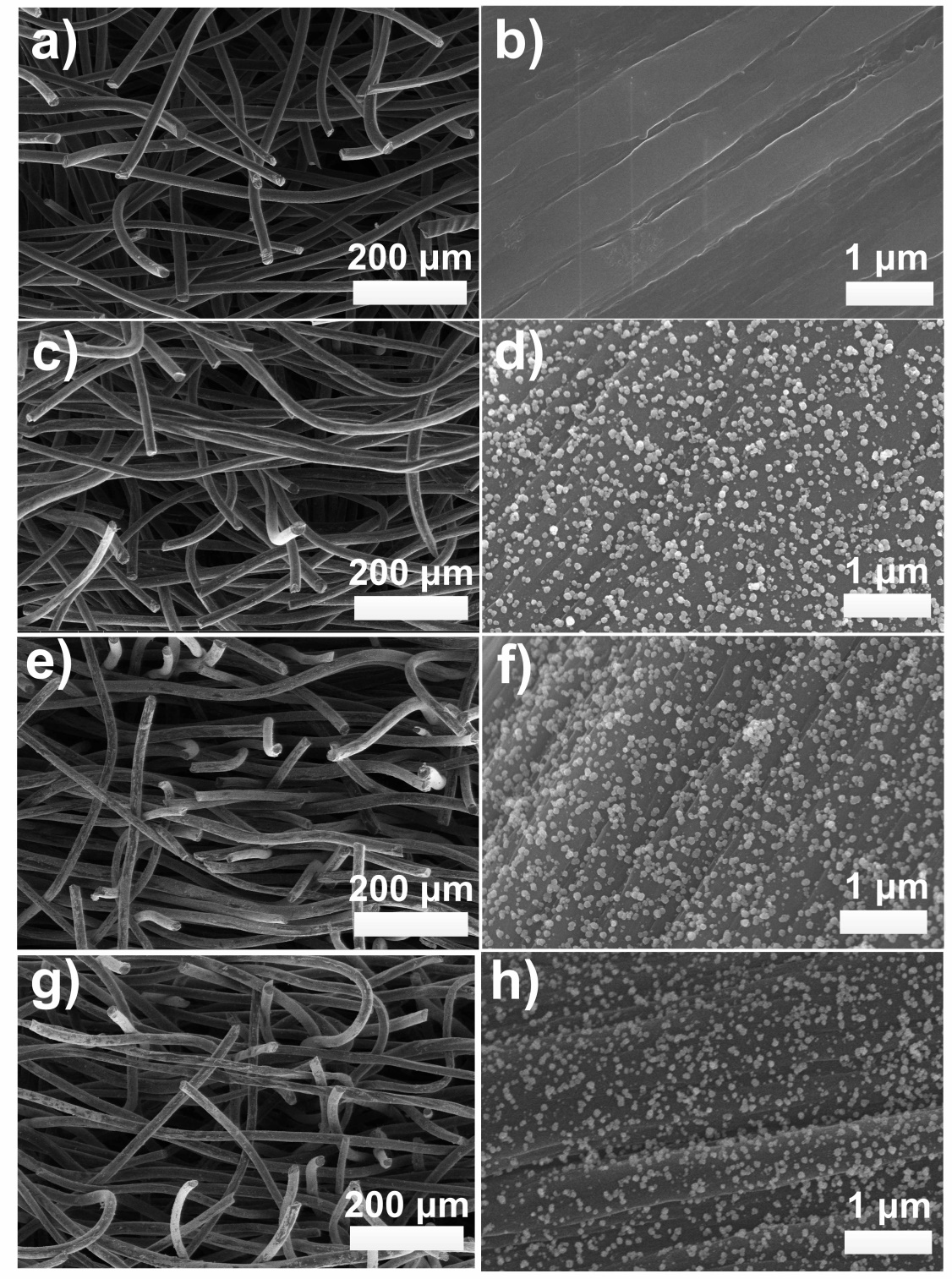


**Supplementary Fig. 8** SEM characterization. SEM images for GF (**a**, **b**), GF-CoS_2_ (**c**, **d**), GF-CoS_2_/CoS (**e**, **f**) and GF-CoS (**g**, **h**).


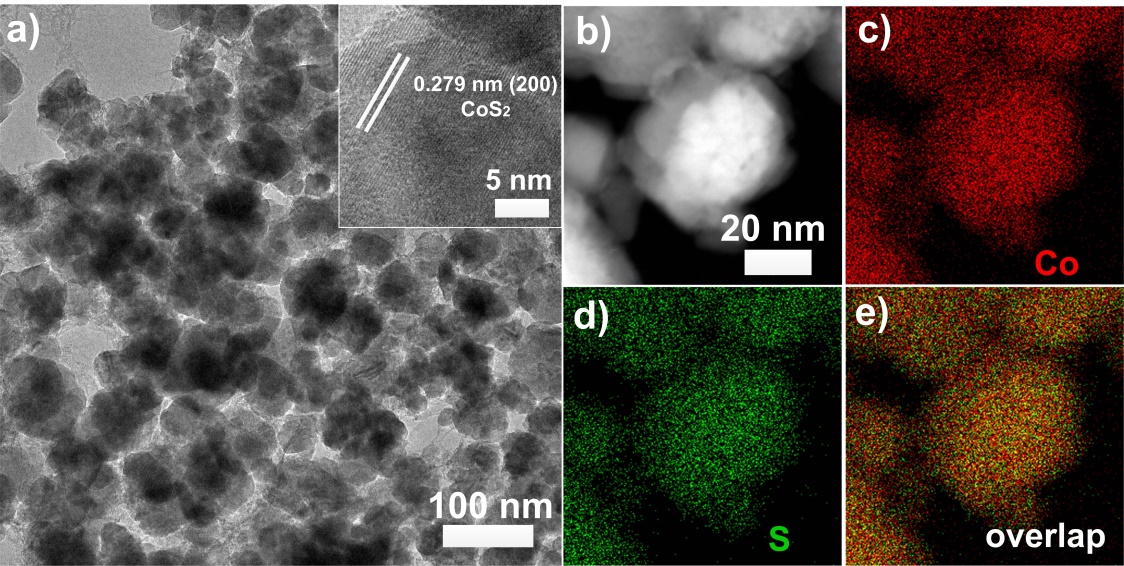


**Supplementary Fig. 9** Morphology characterization. (**a**) TEM image of the as-prepared CoS_2_, the inset shows the HRTEM images with the lattice fringe spacing of 0.279 nm (CoS_2_). (**b-e**) HAADF-STEM image of CoS_2_ and the elemental mappings of Co and S.


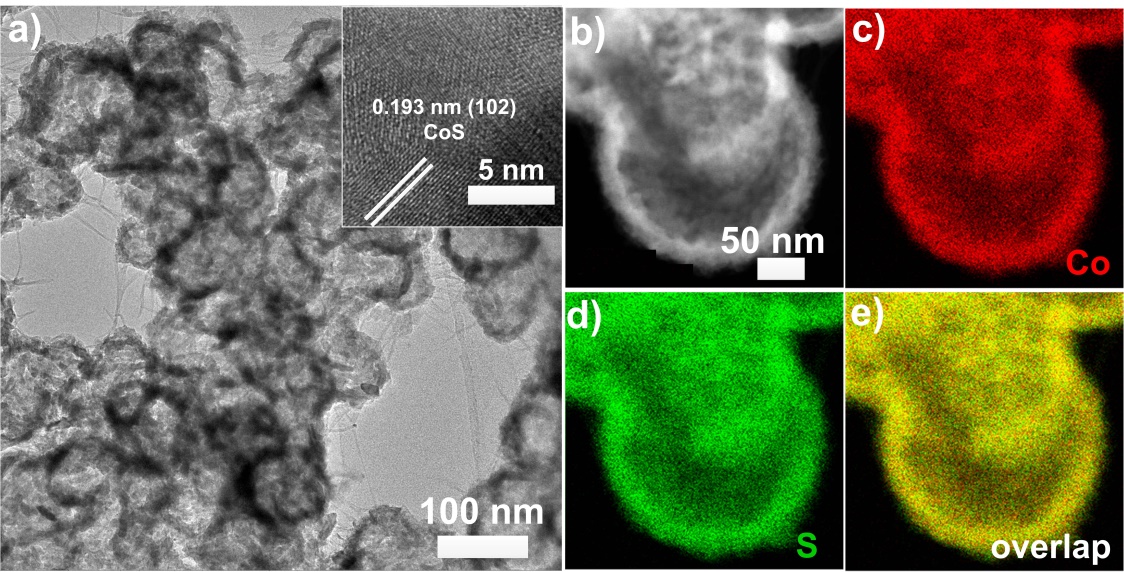


**Supplementary Fig. 10** Morphology characterization. (**a**) TEM image of the as-prepared CoS, the inset shows the HRTEM images with the lattice fringe spacing of 0.193 nm (CoS). (**b-e**) HAADF-STEM image of CoS and the elemental mappings of elements Co and S.

**Supplementary Note 2**

As shown in Supplementary Figs. 9, 10, it was found that the as-synthesized CoS_2_ are nanospheres with size ranging from 50 to 100 nm. HRTEM image reveals the interplanar distance of 0.248 nm and 0.279 nm, corresponding to the (210) plane and (200) plane of CoS_2_. While CoS sample is hollow nanospheres in diameter of 150 to 200 nm with the interplanar distance of 0.193 nm, corresponding to the (102) plane of CoS.


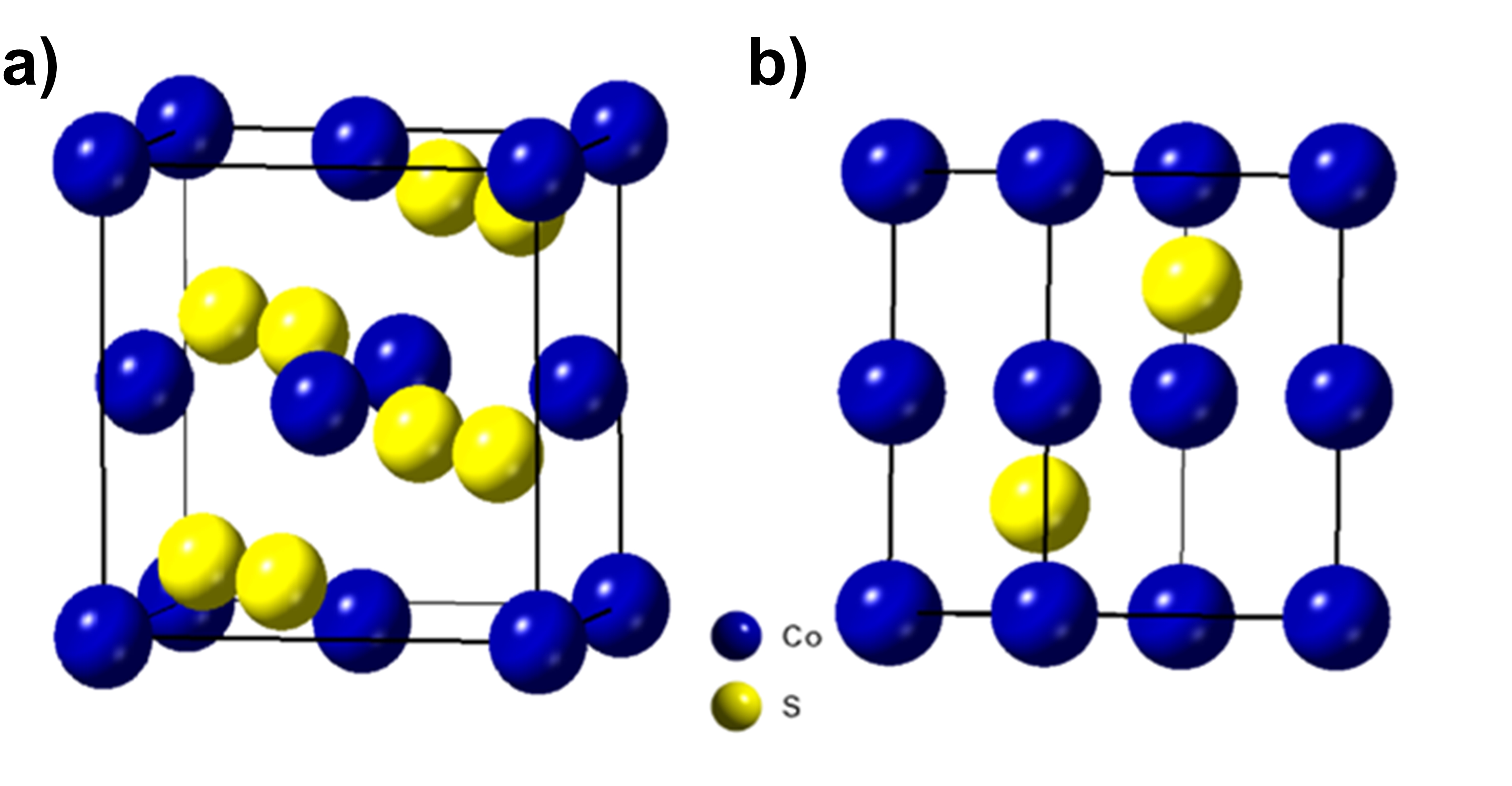


**Supplementary Fig. 11** Structure of materials. The crystal structures of (**a**) CoS_2_ and (**b**) CoS.


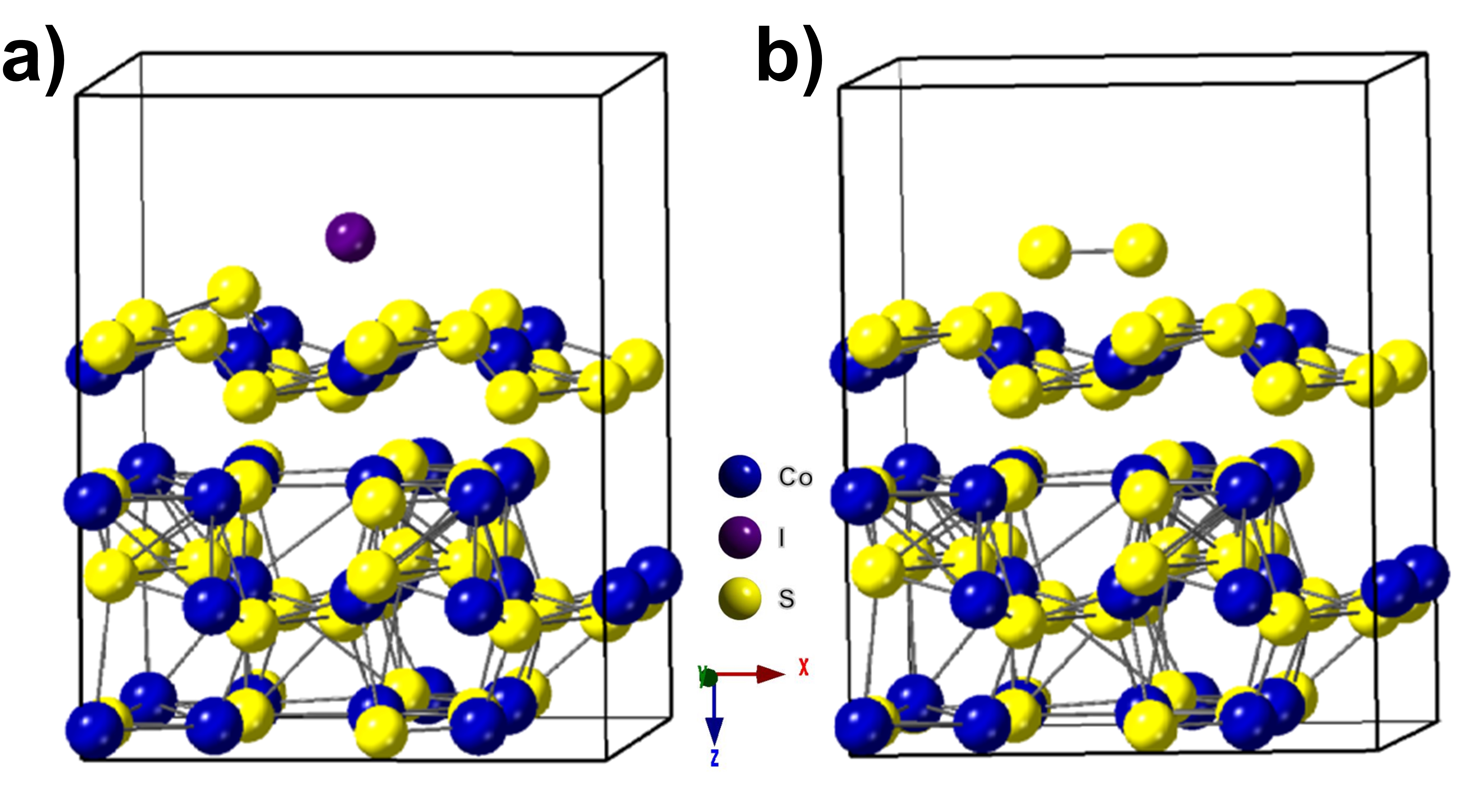


**Supplementary Fig. 12.** Adsorption pattern. The CoS_2_ (200)/CoS (102) heterojunction surface structure, I adsorption (**a**) and S_2_^2-^ (**b**) adsorption.

**Supplementary Note 3**

A bulk of 3D cubic CoS_2_ crystal having a periodicity of Pa3 (205) or P21/A-3 and CoS 3D hexagonal crystal with P63/MMC (194) or P63/M2/M2/C space group is used. The CoS_2_ (2 0 0) and CoS (1 0 2) surface is modeled by cutting the optimized CoS_2_ and CoS bulk crystals along (2 0 0) and (1 0 2) direction, and the thickness of surface slab 1.5 by 2.522 Å is chosen. To build a super cell, a vacuum layer of 15 Å is used along the c direction normal to the surface to avoid periodic interactions with a crystal thickness of 17.1017 Å and super cell range (A=2, B=2 and C=1).^4^


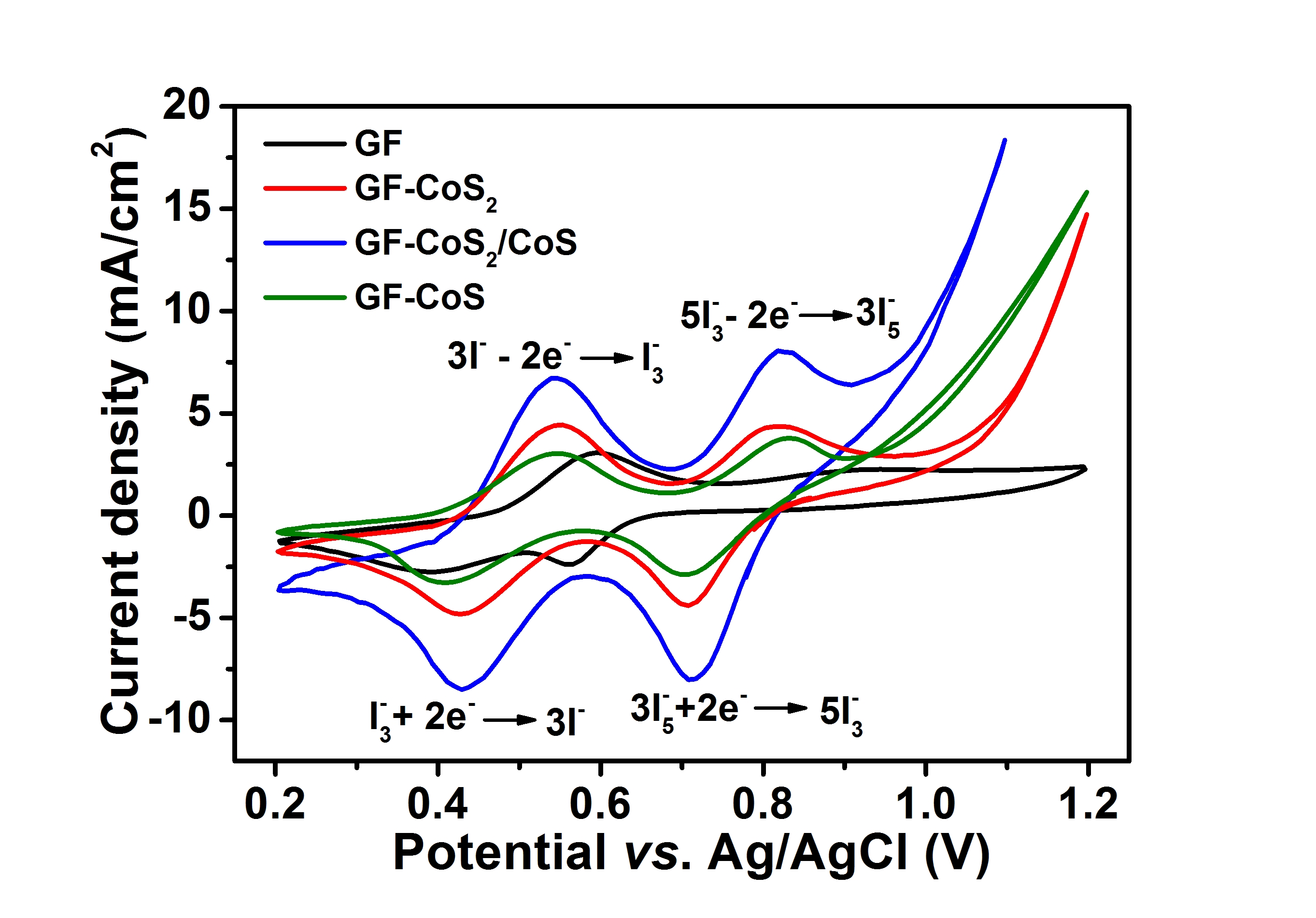


**Supplementary Fig. 13** Cyclic voltammetry characterization. The cyclic voltammograms of GF, CoS_2_, CoS_2_/CoS and CoS electrodes in 4 mM NaI + 0.5 M NaCl solution at a scan rate of 50 mV s^-1^ in the voltage range of 0.2 to 1.2 V *vs.* Ag/AgCl.


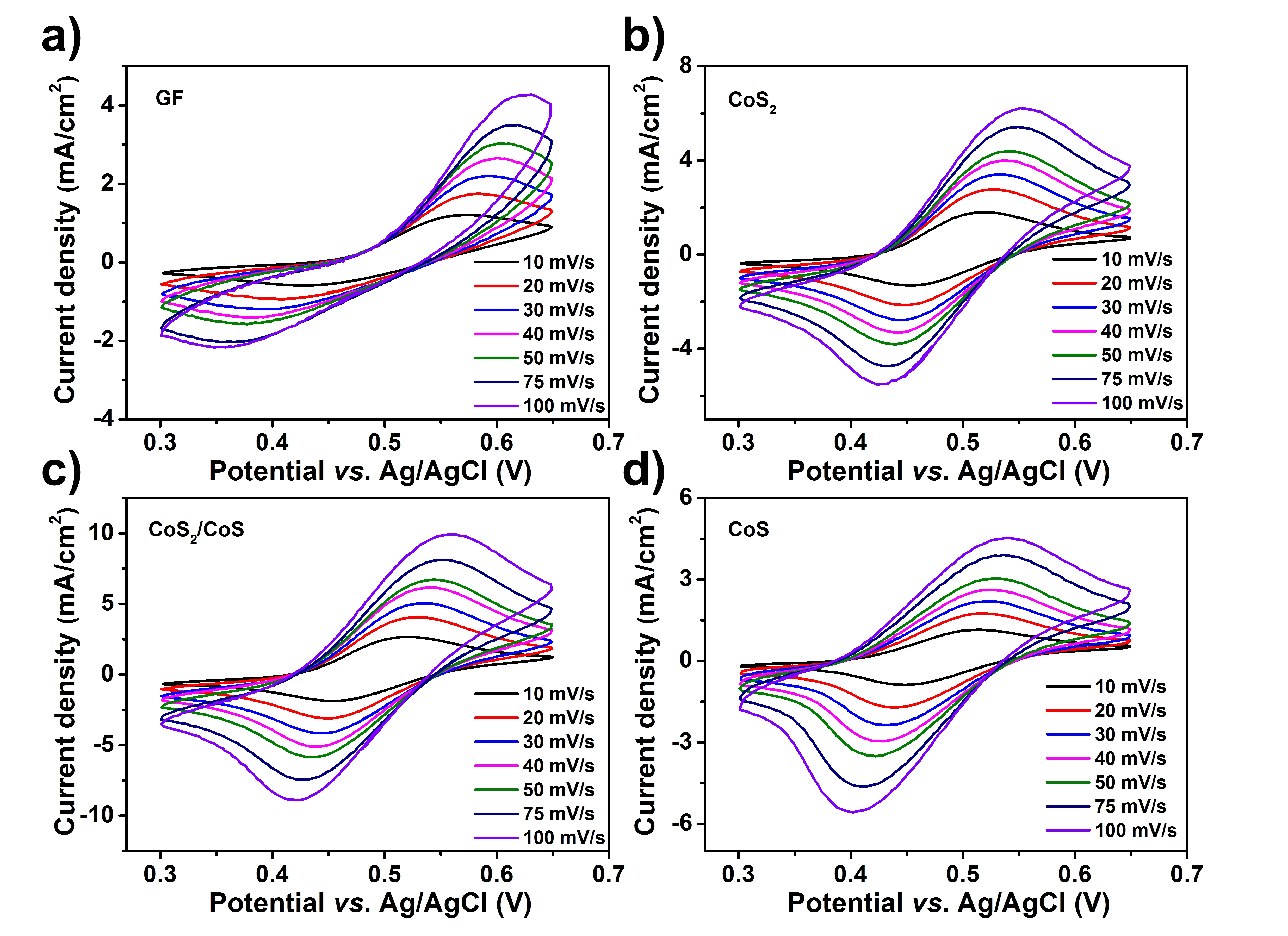


**Supplementary Fig. 14** Cyclic voltammetry characterization. CV curves of GF (**a**), CoS_2_ (**b**), CoS_2_/CoS (**c**) and CoS (**d**) in 4 mM NaI + 0.5 M NaCl solution at various scan rates.


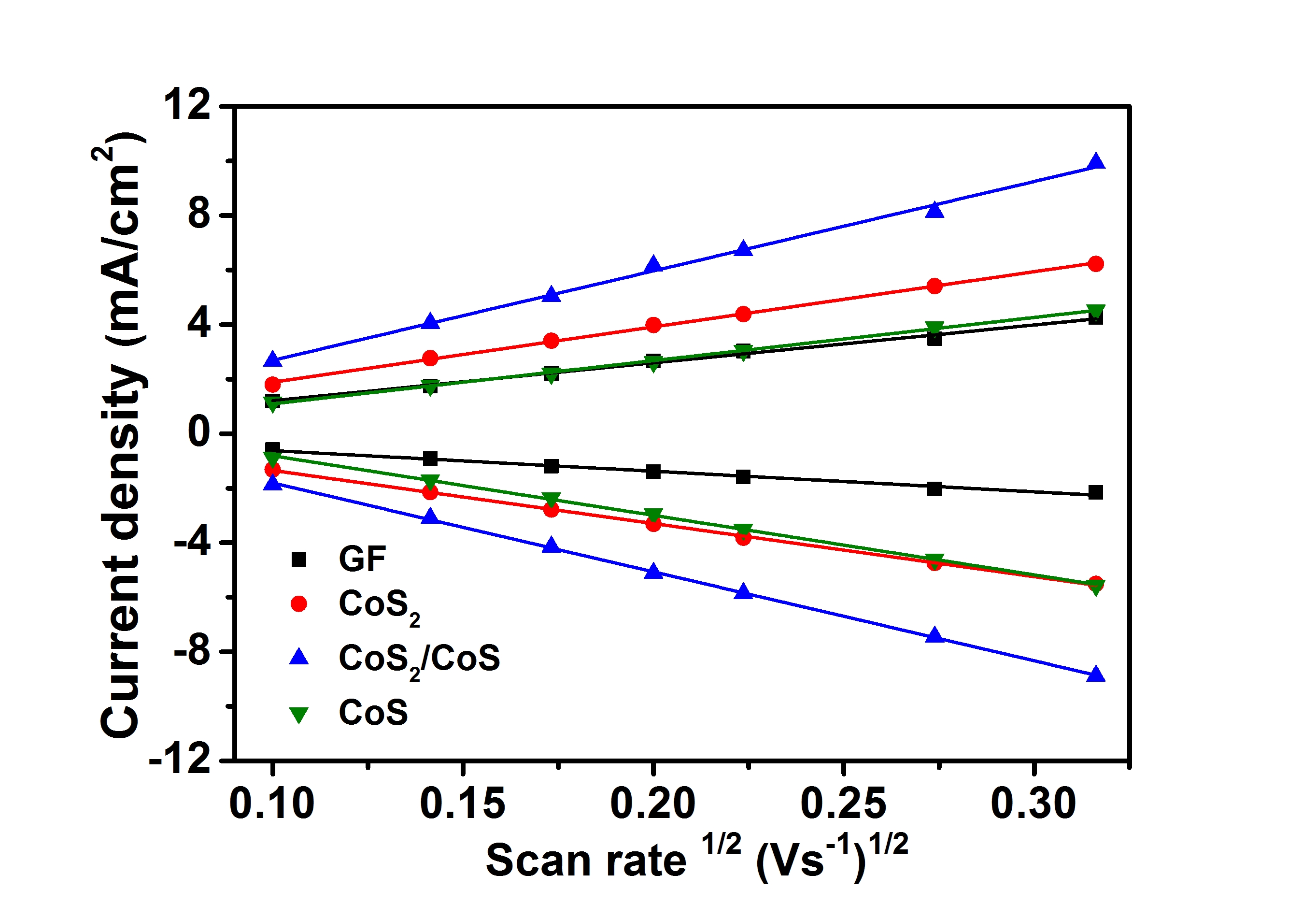


**Supplementary Fig. 15** The curves relationship between the peak current density and the square root of scanning rate based on CV curves of GF, CoS_2_, CoS_2_/CoS and CoS in 4 mM NaI + 0.5 M NaCl solution at various scan rates.

**Supplementary Table 2.** The diffusion coefficient *D* of GF, CoS_2_, CoS_2_/CoS and CoS.

|  | GF | CoS_2_ | CoS_2_/CoS | CoS |
| --- | --- | --- | --- | --- |
| The diffusion coefficient *D* (cm^2^ s^-1^) | 2.85×10^-4^ | 5.99×10^-4^ | 1.41×10^-3^ | 2.89×10^-4^ |

**Supplementary Note 4**

The diffusion coefficient can be estimated by the Berzins-Delahay equation (Equation S2).^5^

*I_p_* = 0.6105(*nF*)^3/2^*SC*_o_*D*^1/2^ν^1/2^(RT)^-1/2^ (Equation S2)

Where *I_p_* is the peak current (A), *n* is the electron transfer number, *S* is the electrode surface area (cm^2^), *C*_o_ is the bulk concentration of I^-^ (mol cm^-3^), *D* is the diffusion coefficient (cm^2^ s^-1^), ν is the potential sweep rate (V s^-1^), *F* is Faraday constant (96485 C/mol). The obtained diffusion coefficient values are listed in Supplementary Table 2.


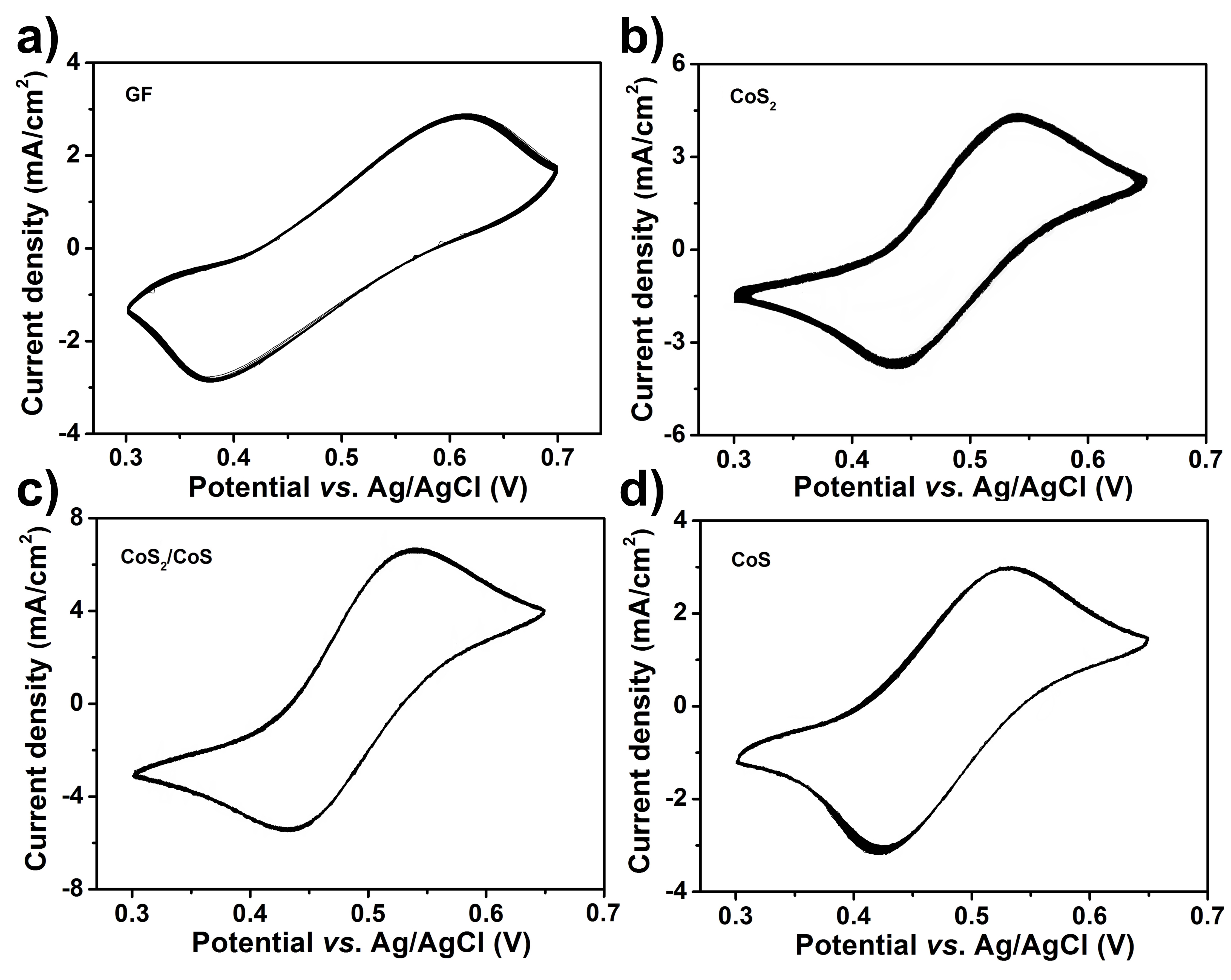


**Supplementary Fig. 16** Cyclic voltammetry characterization. A total of 200 consecutive CV curves of GF (**a**), CoS_2_ (**b**), CoS_2_/CoS (**c**) and CoS (**d**) at a scan rate of 50 mV s^-1^ in 4 mM NaI + 0.5 M NaCl solution.


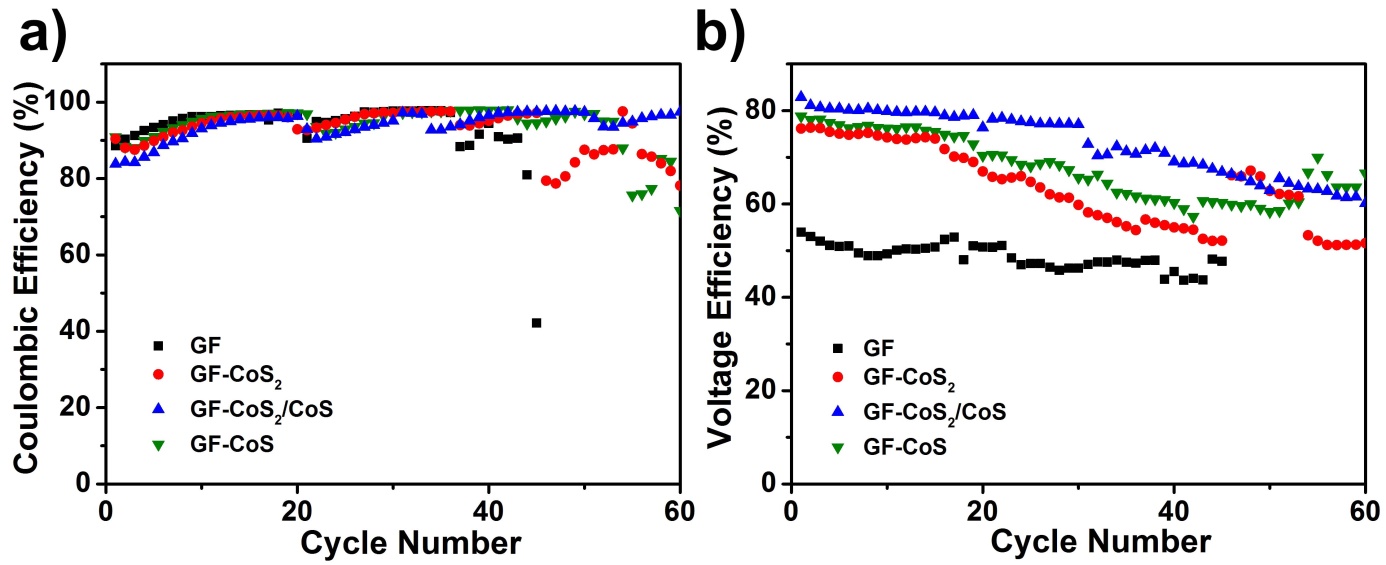


**Supplementary Fig. 17** Performance of the polysulfide/iodide redox flow batteries. The coulombic efficiency (**a**) and voltage efficiency (**b**) of the SIFBs at 20 mA cm^-2^.


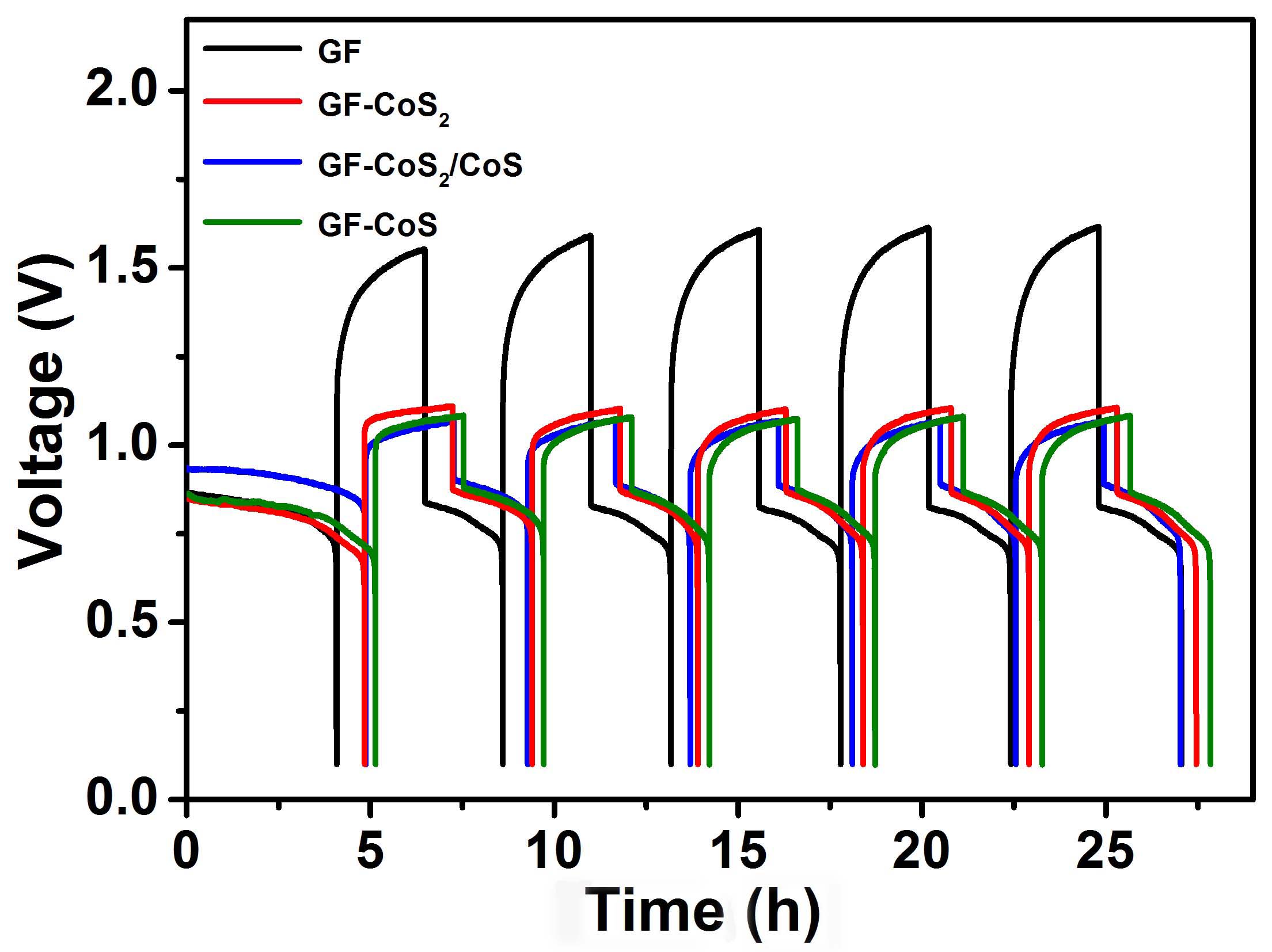


**Supplementary Fig. 18** Performance of the polysulfide/iodide redox flow batteries. Voltage profiles of the SIFBs based on GF, GF-CoS_2_, GF-CoS_2_/CoS, GF-CoS electrodes at 20 mA cm^-2^.


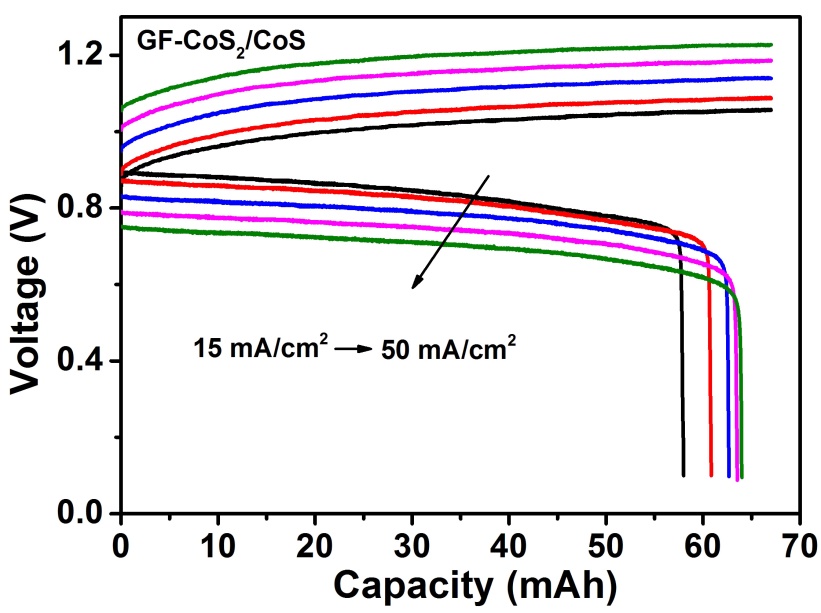


**Supplementary Fig. 19** The rate capability of the polysulfide/iodide redox flow batteries. Representative charge and discharge curves at current densities from 15 mA cm^-2^ to 50 mA cm^-2^ for the SIFB based on GF-CoS_2_/CoS electrode.


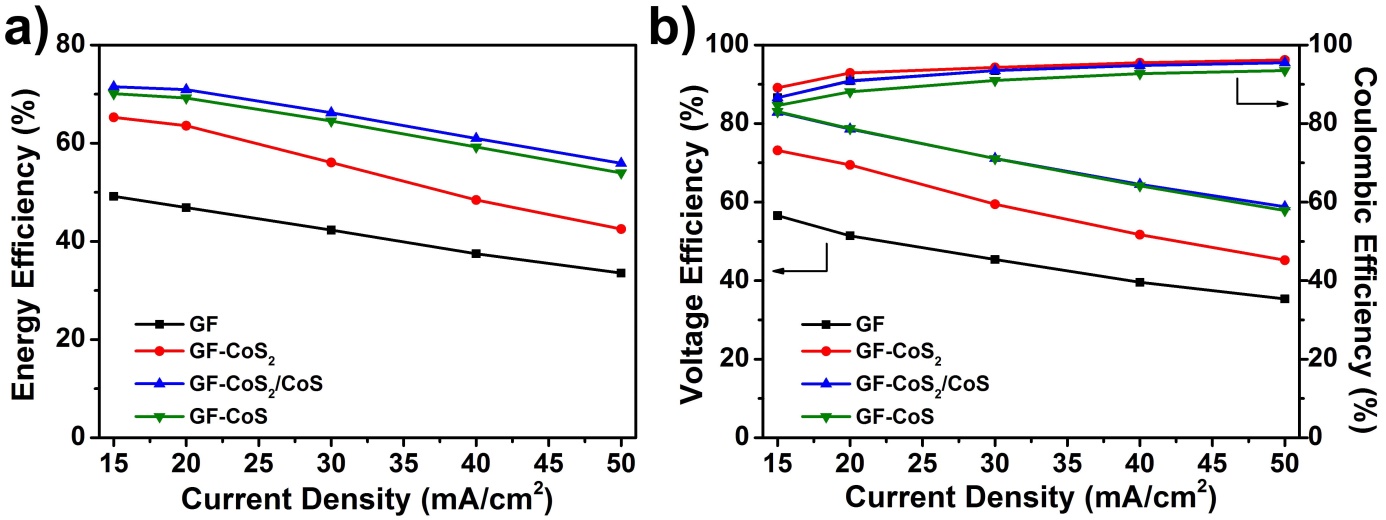


**Supplementary Fig. 20** The rate capability of the polysulfide/iodide redox flow batteries. Plots of average coulombic efficiency (CE), energy efficiency (EE), and voltage efficiency (VE) at different operational current densities.


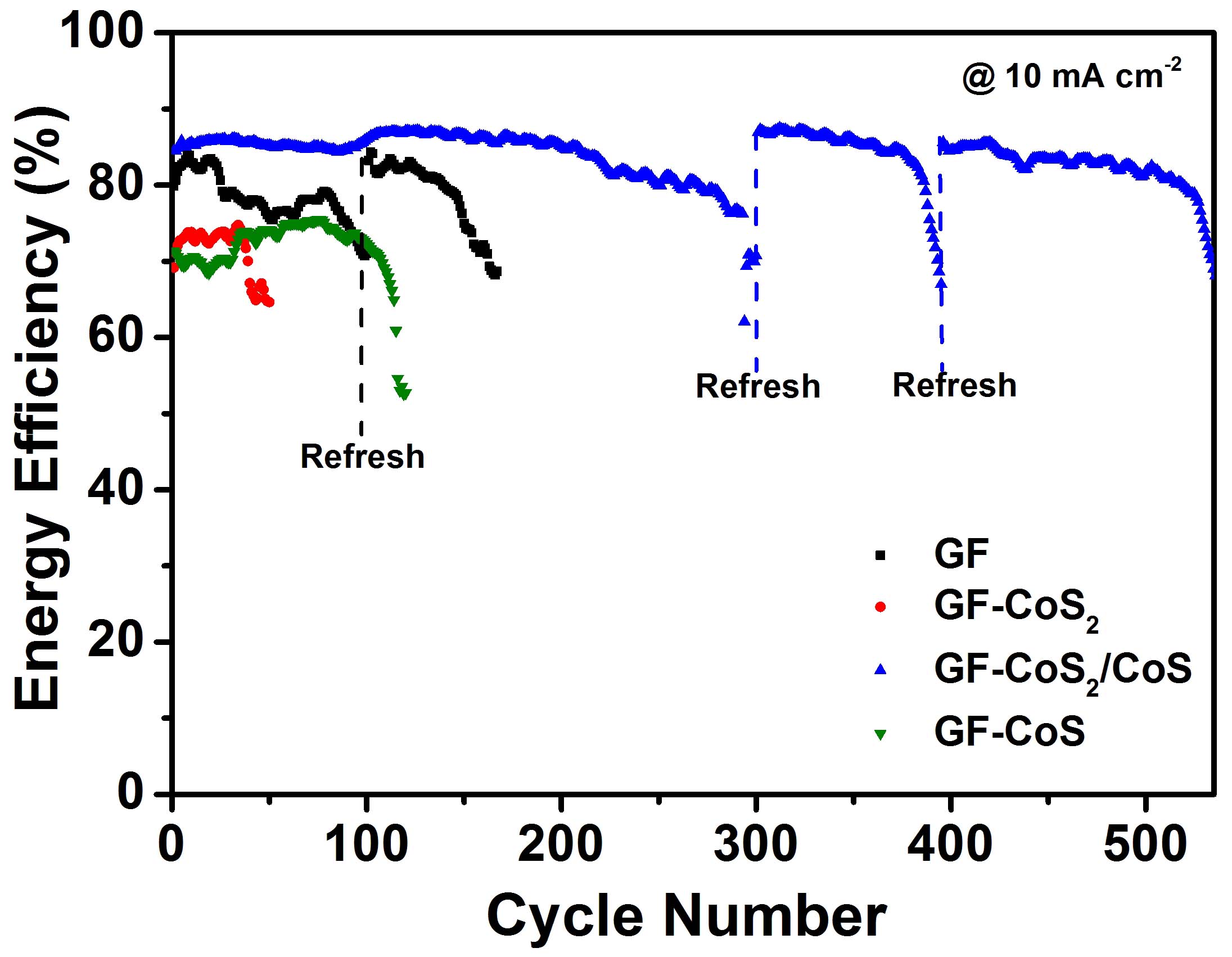


**Supplementary Fig. 21** Performance of the polysulfide/iodide redox flow batteries. The galvanostatic cycling energy efficiency of the SIFBs at 10 mA cm^-2^ with the 10% SOC.


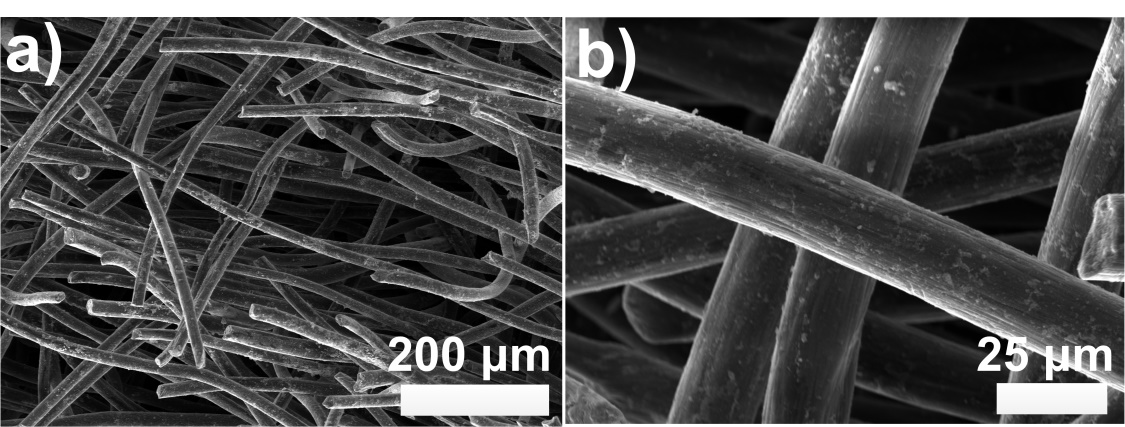


**Supplementary Fig. 22** SEM characterization. SEM images of GF-CoS_2_/CoS electrode after 60 cycles at 20 mA cm^-2^.


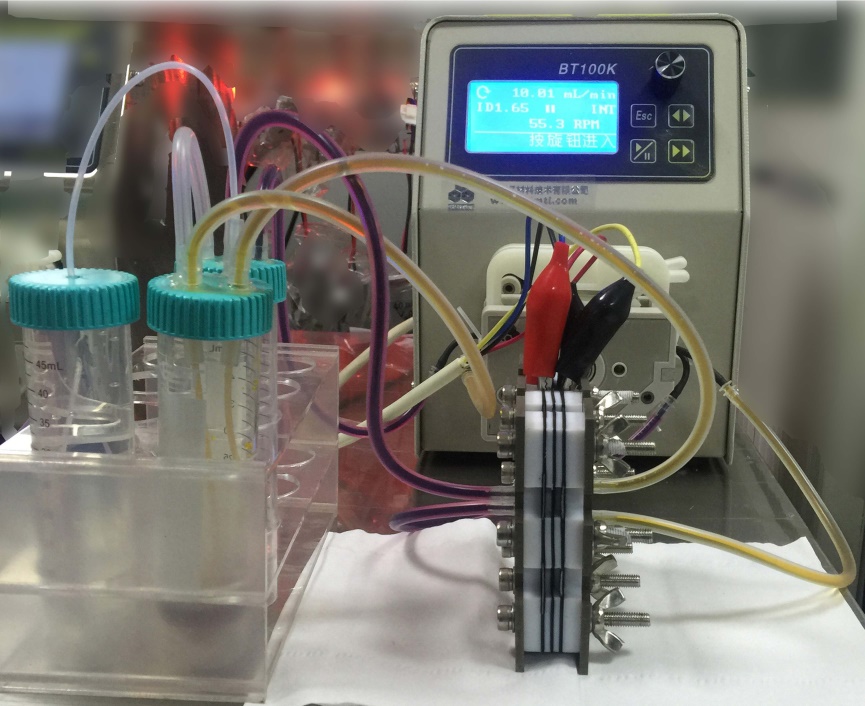


**Supplementary Fig. 23** Configuration of the polysulfide/iodide redox flow batteries. A photograph of the assembled SIFB single cell.

**Supplementary References**

1. Gelderman, K., Lee, L. & Donne, S. W. Flat-Band Potential of a Semiconductor: Using the Mott-Schottky Equation. *J. Chem. Educ.* **84**, 685-688 (2007).
2. Wei, F., Liu, Y., Zhao, H., Ren, X., Liu, J., Hasan, T., Chen, L., Li, Y. & Su, B. L. Oxygen Self-Doped g-C_3_N_4_ with Tunable Electronic Band Structure for Unprecedentedly Enhanced Photocatalytic Performance. *Nanoscale* **10,** 4515-4522 (2018).
3. Xu, M., Ye, T., Dai, F., Yang, J., Shen, J., He, Q., Chen, W., Liang, N., Zai, J. & Qian, X. Rationally Designed n-n Heterojunction with Highly Efficient Solar Hydrogen Evolution. *ChemSusChem* **8**, 1218-1225 (2015).
4. Segall1, M. D., Lindan, P. J. D., Probert, M. J., Pickard, C. J., Hasnip, P. J., Clark S. J. & Payne, M. C. First-principles Simulation: Ideas, Illustrations and the CASTEP code. *J. Phys. Condens. Matter.* **14**, 2717-2744 (2002).
5. Berzins, T. & Delahay, P. Oscillographic Polarographic Waves for the Reversible Deposition of Metals on Solid Electrodes. *J. Am. Chem. Soc.* **75**, 555-559 (1953).
